# Light and ultrasound activated precision: a novel water-soluble BODIPY-mediated sono-photosensitizer in SPDT for breast cancer treatment

**DOI:** 10.1038/s41598-026-48642-9

**Published:** 2026-04-16

**Authors:** Ceren Can Karanlık, Ayşegül Türkkol, Gürkan Karanlık, Umut Kerem Kolaç, Şerife Gökçe Çalışkan, Mehmet Dinçer Bilgin, Ali Erdoğmuş

**Affiliations:** 1 Department of Chemistry, Faculty of Arts and Science, Yildiz Technical University, 34220 Istanbul, Turkey; 2 Department of Biophysics, Faculty of Medicine, Aydin Adnan Menderes University, 09010 Aydın, Turkey; 3 Health Biotechnology Joint Research and Application Center of Excellence, 34220 Istanbul, Turkey; 4 Department of Medical Biology, Faculty of Medicine, Aydin Adnan Menderes University, 09010 Aydın, Turkey; 5 Department of Physics, Faculty of Sciences, Aydın Adnan Menderes University, 09010 Aydın, Turkey

**Keywords:** BODIPY, Combination therapy, SPDT, Breast cancer, Biochemistry, Cancer, Chemical biology, Chemistry, Drug discovery

## Abstract

Remarkable results have been achieved by using the synergistic effect of light and ultrasound in sono-photodynamic therapy (SPDT). This application, which has been defined as a combination treatment method in recent years, aims to increase the amount of singlet oxygen produced by sono-photosensitizers. This research aims to assess the singlet oxygen generation potential of BODIPY compounds through the utilization of the SPDT method. Motivated by this fact, we synthesized and characterized a new water-soluble BODIPY compound doped with a heavy-atom and a distyryl moiety. The singlet oxygen production capacities of the compounds were investigated both photochemically and sono-photochemically. To assess their biological performance, in vitro studies were conducted using the MDA-MB-231 breast cancer cell line. Cytotoxicity and apoptosis were determined by MTT and Annexin V-FITC/PI assays, while ROS generation was detected using DHE staining under confocal microscopy. *MnSOD* and *GPX1* expression levels were analyzed to evaluate mitochondrial antioxidant responses. The results demonstrated that both BODIPY compounds significantly enhanced reactive oxygen species (ROS) generation and apoptosis under SPDT, accompanied by increased *MnSOD* and *GPX1* expression. In addition, molecular docking studies were conducted to evaluate the binding interactions of the newly synthesized compounds with the EGFR target protein, providing insight into their potential as multifunctional agents with both photodynamic and molecular-targeting capabilities. Molecular docking results demonstrated that the newly synthesized compounds possess markedly higher EGFR binding affinity than cisplatin, supported by lower binding energy values and stronger active-site interactions. These findings suggest that the synthesized BODIPY derivatives act as efficient sono-photosensitizers capable of inducing ROS-mediated apoptosis, highlighting their potential as promising agents for cancer therapy. Although BODIPY derivatives are well known as photosensitizers in PDT, their potential as sono-photosensitizers in SPDT has been rarely explored. This study therefore addresses an important gap by assessing the SPDT efficacy of newly synthesized water-soluble BODIPY derivatives in MDA-MB-231 cells.

**Supplementary Information:**

The online version contains supplementary material available at 10.1038/s41598-026-48642-9.

## Introduction

Breast cancer is one of the most frequently diagnosed malignancies among women and remains the second leading cause of cancer-related mortality worldwide^1^.

Standard treatment strategies often involve a combination of surgical resection, chemotherapy, radiotherapy, hormone therapy, and biological agents. Despite advances in early detection through regular screening programs, breast cancer continues to pose a major clinical challenge due to the high recurrence rate, particularly at the tumor bed after breast-conserving surgery (≤ 90%)—and the poor prognosis of certain molecular subtypes^2^. Among these, triple-negative breast cancer stands out as one of the most aggressive and therapeutically resistant forms, as it is defined by the absence of estrogen receptor (ER), progesterone receptor (PR), and human epidermal growth factor receptor 2 (HER2) expression, accounting for approximately 15% of all breast cancer cases and exhibiting limited therapeutic options^3^.

Photodynamic therapy (PDT) has emerged as an alternative or complementary therapeutic strategy in fighting against cancer diseases^4–6^. PDT demonstrates its capacity to selectively and efficiently target and concurrently treat lesions across extensive surface areas, while presenting minimal or no risk of scarring after healing^7,8^. According to the well-established procedure, PDT performs its function through three separately non-toxic components: drug, commonly stated as photosensitizing agent or photosensitizer (PS), suitable wavelength of light and tissue oxygen^9,10^. Photosensitizers are organic dye materials capable of absorbing electromagnetic radiation and transfer its energy to molecular oxygen, resulting in the formation of reactive oxygen species (ROS) such as hydroxyl radicals, hydrogen peroxide, superoxide anion or singlet oxygen (^1^O_2_) that inflict harm to target tumor cells via inducing either apoptotic or necrotic death^11–13^.

Despite a promising therapeutic approach and offers important benefits in patients, PDT still has some challenges to circumvent. Shallow penetration depth of light constrains the ability of PDT to treat deep-seated tumors^14,15^. To overcome this limitation, researchers have turned their focus to developing a more innovative cancer treatment approach called sonodynamic therapy (SDT)^16^, derived from photodynamic therapy. SDT employs low-frequency ultrasound (US) instead of light as the energy source to activate the sensitizers. SDT may offer broader clinical applications by taking advantage of the deeper tissue penetration of ultrasound, thereby eliminating the main disadvantage of PDT^17,18^. Recently, a much more promising method, sono-photodynamic therapy (SPDT), has emerged as a therapeutic combination of photodynamic therapy and sonodynamic therapy. SPDT encompasses the principles of light-driven (PDT) and ultrasound-driven (SDT) methods to eradicate cancer cells including lung cancer, glioblastoma, liver and pancreatic cancers and breast cancers. The dual activation can give superior characteristics to SPDT such as a deeper tissue penetration, reduced toxic side effects, improved cytotoxic effect and higher singlet oxygen formation than PDT and SDT individual therapies^19–25^.

As third-generation sensitizers, boron-dipyrromethenes (BODIPYs)^26^ have gained attention from researchers for their potential in therapeutic applications. They are characterized by high molar extinction coefficients, a unique small Stokes shift, stability to light and environmental factors and long wavelengths of emission as well as high solubility in common organic solvents. Owing to feasible for the functionalization from all reactive sites, the absorption and emission features of BODIPY fluorophores can be precisely tuned. By introducing suitable segments onto the BODIPY framework, remarkable changes on the photophysical properties can be obtained^27–29^. For example, placing distyryl segments at 3- and 5-positions shifts the absorption band to the red or near-infrared (NIR) region^30–32^. From a photochemical perspective, BODIPYs are efficient singlet oxygen generators, making them excellent photosensitizers in therapeutic solutions. Studies have revealed that the addition of heteroaryl moieties or heavy atoms such as iodine or bromine is an efficient way to promote spin-orbital coupling (SOC) and so make easier singlet-to-triplet *intersystem crossing* (ISC) process, resulting in an improvement of the singlet oxygen quantum yields (Φ_∆_) of BODIPYs in therapeutic treatments^33–37^. BODIPY derivatives have attracted considerable attention in cancer research due to their excellent photostability, high molar absorption coefficients, and tunable photophysical properties. Their structural versatility allows chemical modification to improve water solubility, enhanced cellular uptake, and singlet oxygen generation efficiency. For example BODIPY compounds with metal complexes such as Platinum^38,39^, Ruthenium^40,41^ significantly enhance efficacy in photodynamic therapy (PDT). Complexation with heavy metals, facilitates singlet–triplet intersystem crossing (ISC) through heavy-atom interaction, strengthening triplet state formation and consequently significantly increasing singlet oxygen quantum yield. Increased singlet oxygen production makes the core cytotoxic mechanism of PDT more efficient, while the metal center also promotes cellular uptake, subcellular targeting (e.g., mitochondrial accumulation), and photoselective apoptosis. Therefore, metal complexation not only transforms BODIPY derivatives into more potent photosensitizers but also enables the production of more controlled, selective, and highly effective PDT agents^42^. BODIPY-based compounds have been widely explored as photosensitizers in various in vitro cancer models, including breast, lung, and prostate cancer cell lines, demonstrating effective photodynamic therapeutic potential^43–45^. To the best of our knowledge, photodynamic and sonodynamic applications of BODIPY derivatives have been reported in the literature, but in vivo or in vitro SPDT studies of BODIPYs for different cancer treatments are very limited^46,47^.

In the design of effective sonosensitizers, *enhanced charge transfer* is an important research direction to reinforce the sonocatalytic activity of sonosensitizers^48,49^. To enhance intramolecular charge transfer, donor and acceptor segments should be strategically incorporated into the molecular framework to promote efficient electron migration between different units. BODIPY-based systems often lack rational design features that simultaneously promote efficient ROS generation under both excitation modes. For effective SPDT performance, sensitizers must exhibit not only strong light absorption and efficient ISC, but also enhanced intramolecular charge transfer to facilitate ultrasound-induced activation processes. Therefore, the development of multifunctional sensitizers with tailored electronic architectures is essential to overcome these limitations. However, most reported BODIPY-based systems have been primarily optimized for photodynamic therapy and often lack rational design features that simultaneously enable efficient activation under both light and ultrasound irradiation. In particular, achieving a balance between strong light absorption, efficient intersystem crossing, and enhanced intramolecular charge transfer remains a significant challenge for dual-function sensitizers in sonophotodynamic therapy.

To address these limitations, we propose a rational molecular design strategy to address these challenges by constructing a donor–π–acceptor (D–π–A) BODIPY system. A pyridine unit was introduced at the *meso-*position as an electron-accepting group, while N,N-dimethyl substituents at the *3,5-*positions act as strong electron donors, promoting intramolecular charge transfer. In addition, iodine atoms were incorporated at the *2,6-*positions to enhance ISC through the heavy-atom effect, thereby increasing singlet oxygen generation efficiency. This design is expected to improve both photodynamic and sonodynamic responses, enabling more effective dual activation under SPDT conditions. A novel distyryl BODIPY compound (*BD*) and its water-soluble derivative (*Q-BD*) were synthesized and characterized by spectroscopic methods (^1^H-NMR, FT-IR, UV–Vis and Mass). Then, we investigated their photophysical, photochemical and sono-photochemical properties. In addition, the in vitro therapeutic potential of the synthesized BODIPY derivatives was evaluated in MDA-MB-231 triple-negative breast cancer cells. MTT assays identified 5 µM as a non-toxic concentration for treatment. At this dose, the effects of SDT, PDT, and SPDT were assessed. Apoptosis, ROS generation, and antioxidant enzyme (*MnSOD* and *GPX1*) responses were analyzed to elucidate the underlying oxidative stress mechanisms and to demonstrate the dual sensitizing capability of BODIPY derivatives in SPDT. Furthermore, since EGFR is a key molecular target involved in breast cancer progression, molecular docking studies were performed to explore the binding interactions of the newly synthesized BODIPY derivatives with EGFR, thereby providing complementary insight into their additional molecular-targeting potential alongside SPDT activity.

## Experimental

### Synthesis

**Scheme 1:**
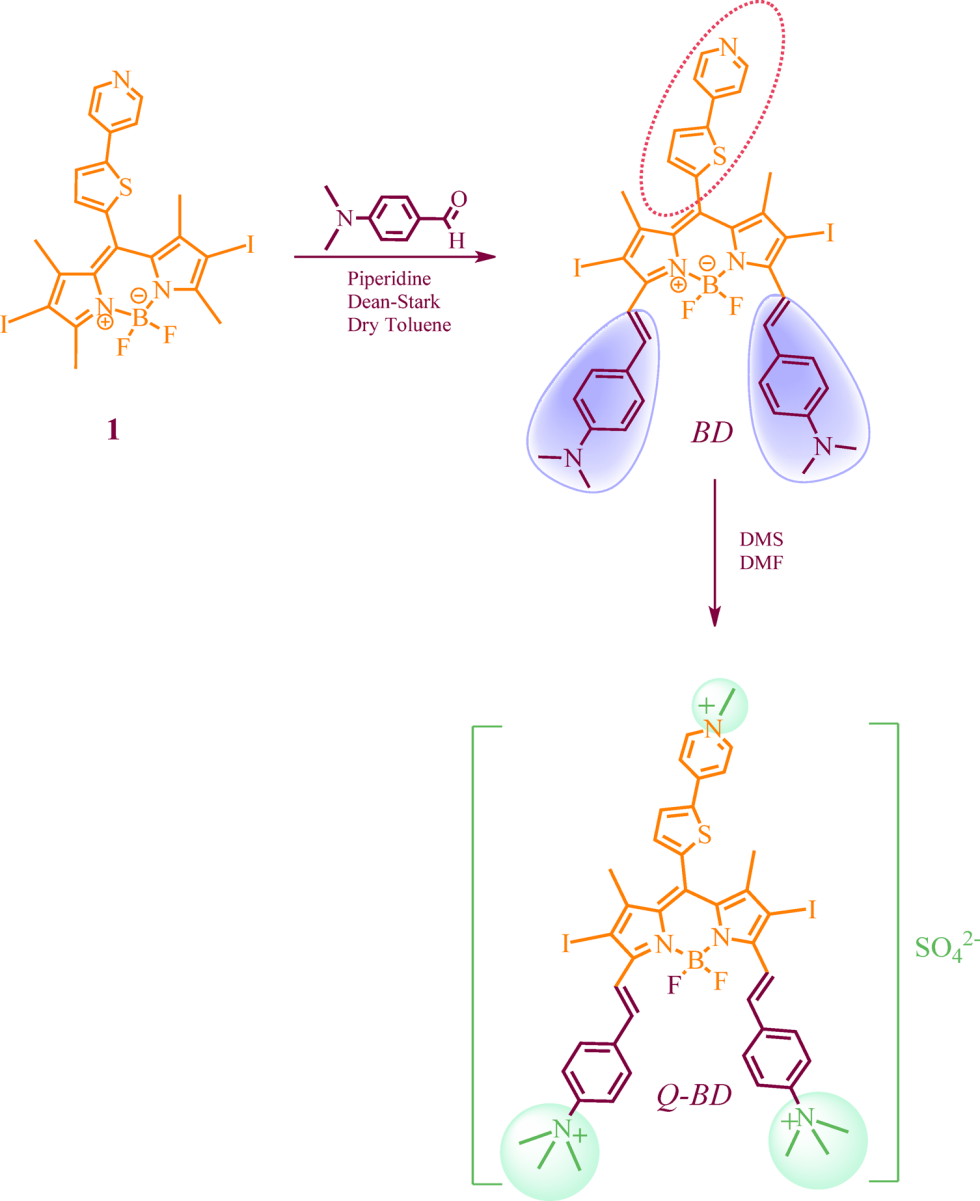
Synthetic pathway for the production of water-soluble distyryl BODIPY derivative *Q-BD*.

The synthetic pathway for the production of novel water-soluble distyryl BODIPY dye *Q-BD* is shown in Scheme 1. The protocol for the synthesis and characterization results of the starting material **1** was given in our previous work^50^. For the synthesis of compound *BD*; precursor **1** and commercially available 4-(dimethylamino)benzaldehyde reacted with each other under Knoevenagel condensation reaction in toluene through Dean-Stark conditions in the presence of piperidine. Then, alkylation of compound *BD* on nitrogen atoms using alkylating agent, dimethyl sulfate (DMS), in DMF gave the target water-soluble distyryl BODIPY derivative *Q-BD*.

The spectroscopic data of intermediate *BD* and of target quaternized BODIPY derivative *Q-BD* were reported for the first time in this study.

#### Synthesis of 4,4-difluoro-2,6-diiodo-1,7-dimethyl-3,5-Bis((E)-4-(dimethylamino)styryl-8-(5-(4-pyridinyl)thiophene-2-yl)-4-bora-3a,4a-diaza-s-indacene (*BD*)

The precursor **1** (0.15 mmol, 0.099 g) was dissolved in dry toluene and a few drops of piperidine were added. After being stirred at 100 °C for 45 min, 4-(dimethylamino)benzaldehyde (0.60 mmol, 0.089 g) was added portionwise to this solution over 2 h under argon atmosphere. The resultant mixture was then stirred for 2 h at 135 °C. The progress of the reaction was checked by thin-layer chromatography (TLC) (CHCl_3_). After the reaction was allowed to cool to room temperature, the solvent was evaporated under reduced pressure. The crude product underwent purification through column chromatography using silica gel 60 eluting with chloroform (CHCl_3_), followed by crystallization from CHCl_3_/MetOH. Yield: 0.049 g (36%). ^1^H-NMR (500 MHz, CDCl_3_): δ (ppm) = 8.66 (d, J ≈ 4.6 Hz; 2H), 8.23 (d, J ≈ 16.5 Hz; 2 olefinic-H), 7.59–7.55 (m, 8H), 7.53–7.52 (m, 3H), 7.06 (d, J ≈ 3.6 Hz; 1 thiophene-H), 6.74 (d, J ≈ 8.5 Hz; 2H), 3.06 (s, 12H, 2 N(CH_3_)_2_), 1.75 (s, 6H, 2 CH_3_). FT-IR (ATR): ʋ_max_(cm^-1^) = 3054.21 (Aromatic C-H), 2980.53, 2915.86 (Aliphatic C–H), 1615.09 (C=N), 1580.36 (C=C), 1392.83 (B-N), 1301.71 (B-F). UV–Vis (DMSO): λ_max_ (nm) = 783. MS (MALDI-TOF): m/z = Calc.: 921.43 g/mol, Found: 921.51 [M]^+^, 795.85 [M^+^-I].

#### Synthesis of water-soluble distyryl BODIPY derivative *Q-BD*

To the distyryl BODIPY derivative *BD* (0.02 mmol, 0.018 g) dissolved in 2 mL of anhydrous DMF, 0.40 mL of dimethyl sulfate (DMS) was added, and the mixture was stirred and boiled under an argon atmosphere at 110 °C for a duration of 24 h. After the reaction was controlled by TLC (CHCl_3_), the mixture was precipitated by pouring into acetone and separated by centrifugation. The water-soluble distyryl BODIPY derivative *Q-BD* was purified by washing with chloroform and *n*-hexane. Yield: 0.014 g (80%). ^1^H-NMR (500 MHz, DMSO-d_6_): δ (ppm) = 8.86 (d, J ≈ 6.5 Hz; 2H), 8.38 (d, J ≈ 6.4 Hz; 2H), 8.34 (d, J ≈ 4.1 Hz; 1H), 8.11 (d, J ≈ 16.6 Hz; 2 olefinic-H), 8.02 (d, J ≈ 8.5 Hz; 4H), 7.87 (d, J ≈ 8.5 Hz; 4H), 7.59 (d, J ≈ 4.1 Hz; 2H), 7.56 (s, 1H), 4.28 (s, 3H, N_pyr_^+^(CH_3_)), 3.62 (s, 18H, 2 N^+^(CH_3_)_3_), 1.75 (s, 6H, 2 CH_3_). FT-IR (ATR): ʋ_max_(cm^-1^) = 3077.35 (Aromatic C-H), 2921.63, 2851.72 (Aliphatic C–H), 1652.21 (C=N), 1581.82 (C=C), 1409.18 (B-N), 1295.48 (B-F). UV–Vis (DMSO): λ_max_ (nm) = 658. MS (MALDI-MS): m/z = Calc.: 1077.63; Found: 1077.34 [M^+^], 1061.63 [M^+^-CH_3_], 950.98 [M^+^-I].

### Procedures applied for photophysical, photochemical and sono-photochemical experiments

To begin, we examined the aggregation behavior of *BD* and *Q-BD* in DMSO. DMSO was chosen for the measurements due to its compatibility, low toxicity, and ability to enhance membrane permeability without causing cellular damage^51^. Aggregation properties of *Q-BD* were also investigated in H_2_O. Moreover, BODIPYs tend to aggregate through strong intermolecular interactions, which can result in either a hypsochromic (blue) or bathochromic (red) shift in the absorption bands of their supramolecular assemblies. To assess this tendency, a series of solutions with increasing concentrations of *BD* and *Q-BD* were prepared, and their absorption spectra were recorded. In addition, to determine their emission characteristics and fluorescence quantum yields, each complex was dissolved in DMSO at fixed concentrations: 1.8 × 10^–5^ M for *BD* and 8.2 × 10⁻^6^ M for *Q-BD*. The detailed methodology for fluorescence quantum yield determination is provided in the Supporting Information.

The singlet oxygen generation capabilities of the complexes were evaluated using two approaches: photochemical and sono-photochemical methods. For both methods, test solutions containing equal amounts of BODIPY (7.2 × 10^–5^ M for *BD* or 3.3 × 10^–5^ M for *Q-BD*) and 1,3-diphenylisobenzofuran (DPBF) (8.5 × 10^−5^ M) were prepared to avoid bright environment. Then, the solutions were exposed to light (7.05 × 10^15^ photons s^−1^ cm^−2^) for photochemical determination. For sono-photochemical determination, test solutions were first exposed to ultrasound (35 kHz), and then to light irradiation. For both determinations, the decrease of DPBF absorbance at 417 nm and ADMA absorbance at 378 nm were pursued using a UV–Vis spectrophotometer.

Photostability assessments of *BD* and *Q-BD* were conducted by preparing their respective solutions in DMSO at concentrations of 7.2 × 10⁻^5^ M for *BD* and 3.3 × 10⁻^5^ M for *Q-BD*. The samples were then subjected to high-intensity light exposure in 10-min intervals to evaluate their stability under photonic stress.

### In vitro studies

#### Cell culture and cytotoxicity analyses

This study aimed to investigate the therapeutic potential of the synthesized compounds as SPDT agents in the human breast adenocarcinoma cell line, MDA-MB-231. The cells were cultured in RPMI-1640 medium (Cat. No. 11875093, GIBCO) supplemented with 10% fetal bovine serum (FBS) (Cat. No. FBS-12A, Capricorn Scientific), 1% Penicillin–Streptomycin (Cat. No. PS-B, Capricorn Scientific), and 1% L-glutamine (Cat. No. GLN-B, Capricorn Scientific). Cultures were maintained at 37 °C in a humidified atmosphere containing 5% CO_2_.

For cytotoxicity analysis, MDA-MB-231 cells were seeded into 24-well culture plates at a density of 1 × 10^5^ cells per well and allowed to adhere overnight. The synthesized compounds were then administered at final concentrations ranging from 1 to 80 µM, followed by incubation for 24 h under dark conditions. After incubation, cell viability was determined using the MTT assay (Invitrogen™, Cat. No: M6494, USA) to assess the dark cytotoxicity of the compounds and to identify the non-toxic concentrations suitable for SPDT application.

For biological assays, the compounds were prepared from DMSO stock solutions and diluted in culture medium. The final concentration of DMSO in the working solutions was 0.05% (v/v). Vehicle control groups containing the same DMSO concentration were included in all experiments, and no solvent-related cytotoxic effects were observed.

#### Ultrasound and light application

For the SPDT experiments, MDA-MB-231 cells were divided into ten groups: control, ultrasound + light, *BD* (5 µM) only, *Q-BD* (5 µM) only, *BD* + SDT, *BD* + PDT, *BD* + SPDT, *Q-BD* + SDT, *Q-BD* + PDT, and *Q-BD* + SPDT. The cells were initially incubated with *BD* and *Q-BD* compounds at a final concentration of 5 µM for 4 h under dark conditions to allow cellular uptake. After incubation, the cells were gently washed with phosphate-buffered saline (PBS) to remove unbound compounds.

Ultrasound irradiation was performed using a BTL Sono device (Model 5710 SONO, BTL, USA) operating at a frequency of 1 MHz, 50% duty cycle, and an acoustic intensity of 0.5 W/cm^2^ for 1 min, corresponding to the stable cavitation level. Ultrasound was applied from the bottom of the culture plate through an 8 cm thick distilled water layer used as the ultrasound transmission medium. In our previous study, it was demonstrated that the ultrasound power density did not show a significant attenuation after propagation through this transmission distance^52^. Immediately after ultrasound exposure, cells were subjected to light irradiation for 5 min using a solar simulator (ABET Solar Simulator, USA) equipped with long-pass (> 600 nm) and short-pass (< 800 nm) filters at a power density of 0.5 mW/cm^2^. Light irradiation was applied from the top of the 6-well plate without any additional spacing between the light source and the well surface (Fig. S7).

Following treatment, all groups were incubated for an additional 24 h under standard culture conditions (37 °C, 5% CO_2_), and cell viability was subsequently determined using the MTT assay.

#### Determination of intracellular ROS

To assess intracellular ROS generation, MDA-MB-231 cells were treated with *BD* and *Q-BD* compounds under the previously described SPDT conditions. Following treatment, cells were incubated with dihydroethidium (DHE) (Invitrogen; Catalog No: D11347), a ROS-sensitive fluorescent probe, according to the manufacturer’s instructions^53^.

For quantitative analysis, fluorescence intensity was measured by flow cytometry using a NovoCyte Flow Cytometer (Agilent, USA). Cells were treated with 100 µM TBHP for 8 h as a positive control for ROS generation.

In parallel, fluorescence signals were visualized using a confocal laser scanning microscope (LSM900, Zeiss, Germany). Images were acquired under identical settings to allow quantitative comparison between experimental groups. The fluorescence intensity of the captured images was analyzed using ImageJ software (NIH, USA), and the results were expressed as mean fluorescence intensity (MFI) values.

#### Determination of apoptotic cell death rates

To determine apoptotic cell death rates, an Annexin V-FITC/PI apoptosis detection kit (Cat No: A026, ABP Bioscience) was used. After 24 h of treatment, the cells were collected and stained according to the manufacturer’s instructions. The percentages of early and late apoptotic cells were quantified using flow cytometry (NovoCyte Flow Cytometer System (Agilent, USA)).

#### Real-time PCR

Total RNA was isolated from MDA-MB-231 cells using the RiboEx RNA Isolation Solution (GeneAll, Cat. No. 301-001, Republic of Korea) according to the manufacturer’s protocol. The concentration and purity of RNA were determined using a NanoDrop spectrophotometer (Micro Digital, Nabi). Complementary DNA (cDNA) was synthesized from 100 ng/µL of total RNA with the WizScript™ cDNA Synthesis Kit (Cat. No. W2211, Republic of Korea). Quantitative real-time PCR (qRT-PCR) analysis was performed using WizPure™ qPCR Master Mix (SYBR; Cat. No. W1711R-5) on a CFX Real-Time PCR System (Bio-Rad, USA). Primer sequences are listed in Table S1. Relative mRNA expression levels were calculated using the 2^−ΔΔCT^ method, with *β-actin* serving as the internal control.

#### Statistical analysis

All experiments were performed in triplicate, and data are presented as mean ± standard error of the mean (SEM). Statistical analyses were conducted using GraphPad Prism 9 (GraphPad Software, USA). The Kolmogorov–Smirnov test was applied to evaluate data normality. For normally distributed data, one-way ANOVA followed by Tukey’s post hoc test was used to determine statistical significance, while non-normally distributed data were analyzed using the Kruskal–Wallis test. A p-value of less than 0.05 was considered statistically significant. IC_50_ values were determined from dose–response curves obtained using nonlinear regression analysis. The sono-phototherapeutic index (SPI) was calculated as the ratio of IC_50_ (dark) to IC_50_ (SPDT), representing the therapeutic selectivity of the compounds under sonophotodynamic treatment conditions^54^.

### Molecular docking analysis

In this study, we investigated the binding potential of two newly synthesized compounds, named *BD* and *Q-BD*, against the Epidermal Growth Factor Receptor (EGFR) using molecular docking techniques. To provide a comparative reference, cisplatin, a widely used chemotherapeutic agent, was included as a positive control.

The three-dimensional crystal structure of EGFR (PDB ID: 1M17) was obtained from the Protein Data Bank. Prior to docking, the protein was prepared using AutoDock Tools 1.5.6 by removing water molecules and its co-crystallized ligand, adding polar hydrogens, and assigning Gasteiger charges.

The 2D structures of *BD* and *Q-BD* were created using ChemSketch, then converted into 3D conformations and geometry-optimized by Avogadro software^55^. The structure of cisplatin (PubChem CID: 5,460,033) was downloaded from the PubChem database and processed using the same steps. All ligands were checked for appropriate protonation states and converted into the PDBQT format required by AutoDock.

Docking simulations were performed with AutoDock 4.2^56^. A grid box measuring 80 Å × 80 Å × 80 Å was defined to fully enclose the active site of the target protein, with its center set according to the co-crystallized ligand in the 1M17 structure. The docking protocol used the Lamarckian Genetic Algorithm (LGA), and each ligand was run 200 times to ensure reliable results. Binding affinities were assessed based on predicted binding free energies (in kcal/mol), and the best-scoring poses were selected for further analysis.

To better understand the interactions, the top docking poses were visualized and analyzed using Discovery Studio Visualizer and PyMOL. Key interactions such as hydrogen bonding, hydrophobic contacts, and electrostatic forces were identified and interpreted to provide insight into the binding mechanisms.

Moreover, the validation of the molecular docking protocol was performed using a self-docking approach, whereby the co-crystallized ligand was re-docked into the active site of the target protein. The root-mean-square deviation (RMSD) between the docked and crystallographic poses was calculated.

## Results and discussion

### Structural characterization

In this study, a novel water-soluble distyryl BODIPY derivative (*Q-BD*) bearing 4-pyridinylthiophene moiety at *meso*- position and heavy iodine atoms at 2- and 6- positions was synthesized. The intermediate *BD* and target derivative *Q-BD* were purified by column chromatography or recrystallization methods and their structures were identified by conventional spectroscopic methods (^1^H-NMR, FT-IR, UV–Vis and MS). In the ^1^H-NMR spectrum of compound *BD* (see Fig. S1), aromatic protons as well as olefinic protons appear at between 8.66 and 6.74 ppm. The doublet peak at 8.23 ppm with a J value of 16.5 Hz. belongs to olefinic hydrogens in the distyryl structure. The doublet peak observed at 7.06 ppm with the coupling constant of 3.6 Hz. represents a proton in the thiophene ring. The singlet signal resonating at 3.06 ppm with an integration value of 12 belongs to the methyl hydrogens in two -N(CH_3_)_2_ substituents. The other singlet signal appearing at 1.75 ppm indicates the methyl protons at 1- and 7- positions of the molecule. ^1^H-NMR spectrum of target water-soluble distyryl BODIPY derivative *Q-BD* (see Fig. S4) is similar to the ^1^H-NMR spectrum of compound *BD* except that the strong evidence of quaternization of nitrogen atoms. The protons in methyl groups attached to the nitrogen atoms -both in the pyridine ring and in the dimethylamino segments- are observed as singlet peak at 4.28 ppm with integration value of 3, corresponding to one methyl group in the methylpyridinium unit, and as a singlet at 3.62 ppm with an integration value of 18, attributed to six methyl groups in the two N^+^(CH₃)_3_ units. In the FT-IR spectrum of compound *BD* (see Fig. S2), aromatic C-H stretching vibration is observed at 3054.21 cm^-1^ and aliphatic C-H stretching vibrations are shown at 2980.53 cm^-1^ and 2915.86 cm^-1^. Additionally, B-N and B-F vibrational bands at 1392.83 cm^-1^ and at 1301.71 cm^-1^, respectively, are characteristic for BODIPY core systems. In the FT-IR spectrum of target water-soluble distyryl BODIPY derivative *Q-BD* (see Fig. S5), aromatic C-H stretching vibration is observed at 3077.35 cm^-1^ and aliphatic C-H stretching vibrations are shown at 2921.63 cm^-1^ and 2851.726 cm^-1^. Vibrational bands shown at 1409.18 cm^-1^ and 1295.48 cm^-1^ represent the B-N and B-F stretching, respectively. In the Mass spectra of *BD* and *Q-BD* (see Figs. S3 and S6), dithranol (DIT) for compound *BD* and α-cyano-4-hydroxycinnamic acid (α-CHCA) for compound *Q-BD*, were used as matrix. Molecular ion peak of compound *BD* was observed at 921.51 [M]^+^ and other fragments were seen at 795.85 [M^+^-I]. On the other hand, molecular ion peak of compound *Q-BD* occurred at 1077.34 [M^+^] and other fragments appeared at 1061.63 [M^+^-CH_3_] and 950.98 [M^+^-I]. These results indicate that molecular weights of the compounds are in accordance with the suggested structures. Considering all data obtained from the ^1^H-NMR, FT-IR and Mass spectra for both compound *BD* and target water-soluble distyryl BODIPY derivative *Q-BD*, it can be concluded that the related compounds were synthesized successfully.

### Optical properties

#### Ground state properties, emission spectra and fluorescence quantum yields

**Fig. 1:**
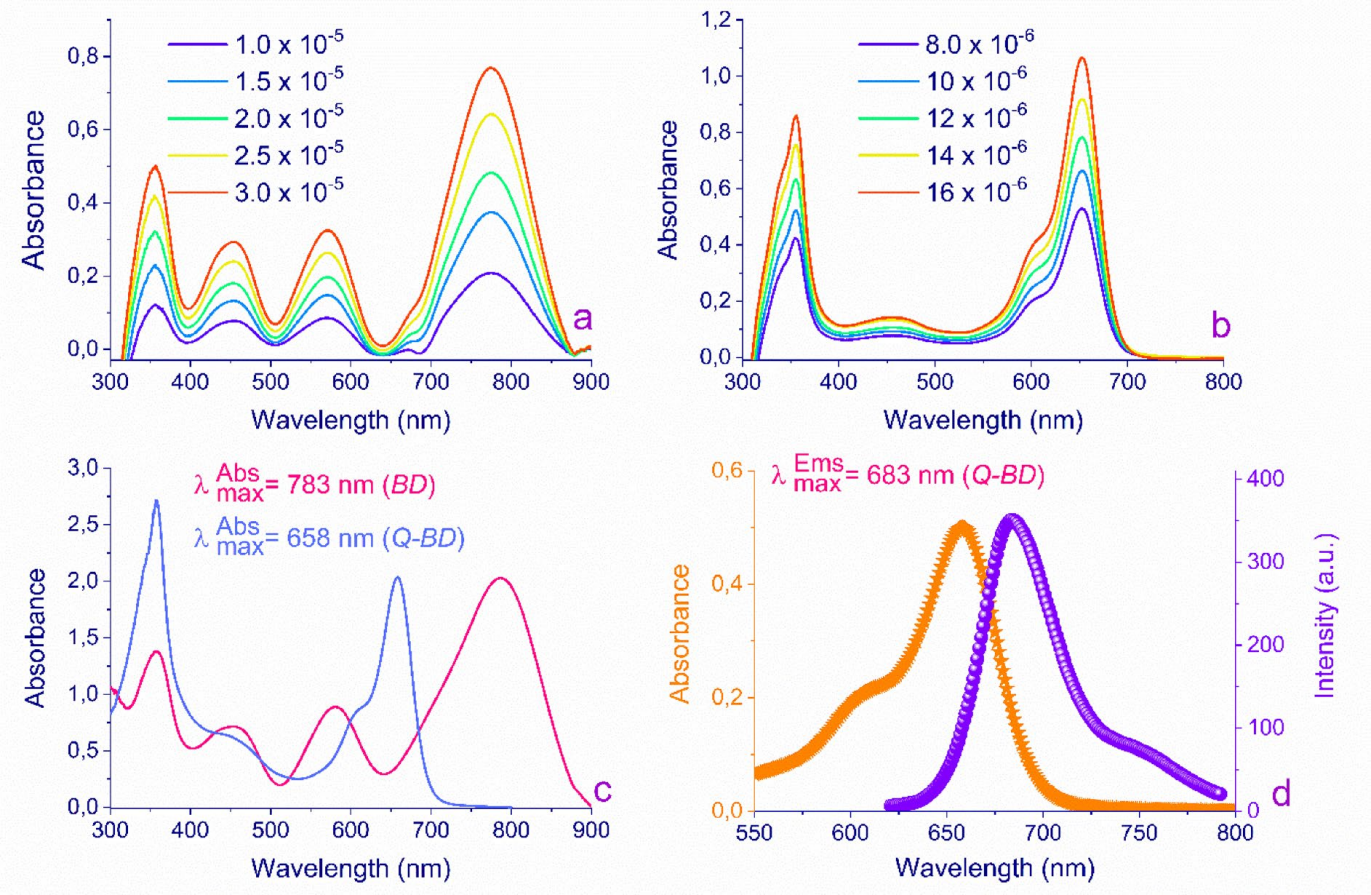
(**a**) UV–Vis dilution studies of *BD* in DMSO, (**b**) UV–Vis dilution studies of the *Q-BD* in DMSO, (**c**) Superimposed UV–Vis spectrum of *BD* and *Q-BD* in DMSO and (**d**) Absorption (658 nm) and emission (683 nm) spectra of *Q-BD* in DMSO.

The absorption spectra of the solutions of the compounds prepared in DMSO at different concentrations were taken and the aggregation properties were examined. Consequently, ε value was calculated as 27,736 M^-1^ cm^-1^ for *BD* and 60,316 M^-1^ cm^-1^ for *Q-BD*. Figure 1a, b includes the UV–Vis dilution studies of *BD* and *Q-BD*, respectively. In addition, the superimposed spectra of *BD* and *Q-BD* are given in Fig. 1c. Maximum absorbances of *BD* and *Q-BD* were observed at 783 nm and at 658 nm, respectively. There is a significant hypsochromic effect between *BD* and *Q-BD*. This is due to the quaternization of the *N,N*-dimethyl segments in the chemical structure of *BD*.

The fluorescence characteristics of compounds are crucial for enabling their detection and traceability within biological systems. However, *BD* does not emit in DMSO. The chemical structure of *BD* enables a pull–push effect. This effect induces *intramolecular charge transfer* abbreviated as ICT. The peaks observed at between 300 and 500 nm in the absorption spectrum of *BD* are evidence for S_0_-S_2_ transitions involving partial ICT which are typically characterized as ‘weak emitters’ and are prone to non-radiative decay via charge recombination, which can lead to partial quenching of the fluorescence in the acceptor moiety. On the other hand, the quaternization of *N*,*N*-dimethyl segment leads to the inhibition of the ICT effect and provides emission for *Q-BD*. Figure 3d contains superimposed spectra of absorption and emission of *Q-BD*. Maximum emission peak was measured at 863 nm with 25 nm of Stokes shift. Also, fluorescence quantum yield of *Q-BD* was calculated as 0.08 in DMSO. All optical values are listed in Table 1.

**Table 1:** Photophysical and photo-sono-photochemical properties of *BD* and *Q-BD* in DMSO.

	λ_Abs_ (nm)	λ_Ems_ (nm)	ε × 10^4^ (M^−1^ cm^−1^)	Φ_f_	Φ_∆PDT_	Φ_∆SPDT_	Φ_d_ (× 10^−4^)
*BD*	783	–	2.77	–	0.24	0.86	3.01
*Q-BD*	658	683	6.31	0.08	0.67/0.42*	0.92	8.03

*In H_2_O.

### Evaluation of photochemical properties

#### Assessment of singlet oxygen generation by photochemically and sono-photochemically

The key focus of this study was to demonstrate how the combined application of ultrasound and light influences singlet oxygen production. A critical property of sensitizer compounds utilized in photodynamic therapy (PDT) and related applications is their efficacy in generating singlet oxygen. Therefore, the choice of sensitizer is crucial in these applications. Incorporating heavy atoms through structural modifications or using heterocycles, particularly thiophene segments, significantly enhances singlet oxygen production^57–59^. Moreover, recent studies have underscored the importance of the methods employed to excite the sensitizer^23,30,60–62^.

**Fig. 2:**
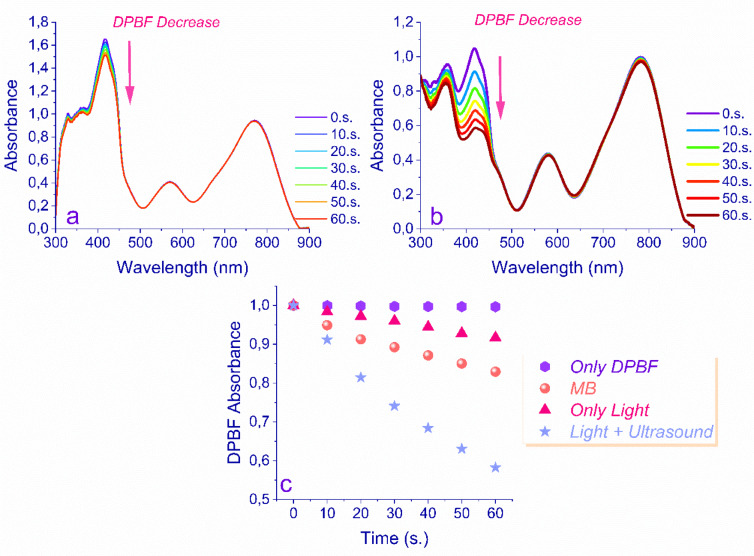
(**a**) Determination of singlet oxygen production for *BD* in DMSO via photochemical pathway, (**b**) Determination of singlet oxygen production for *BD* in DMSO via sono-photochemical pathway, (**c**) Time-dependent decrease of absorbance at 417 nm by oxidation of DPBF with *BD* under light and ultrasound + light.

**Fig. 3:**
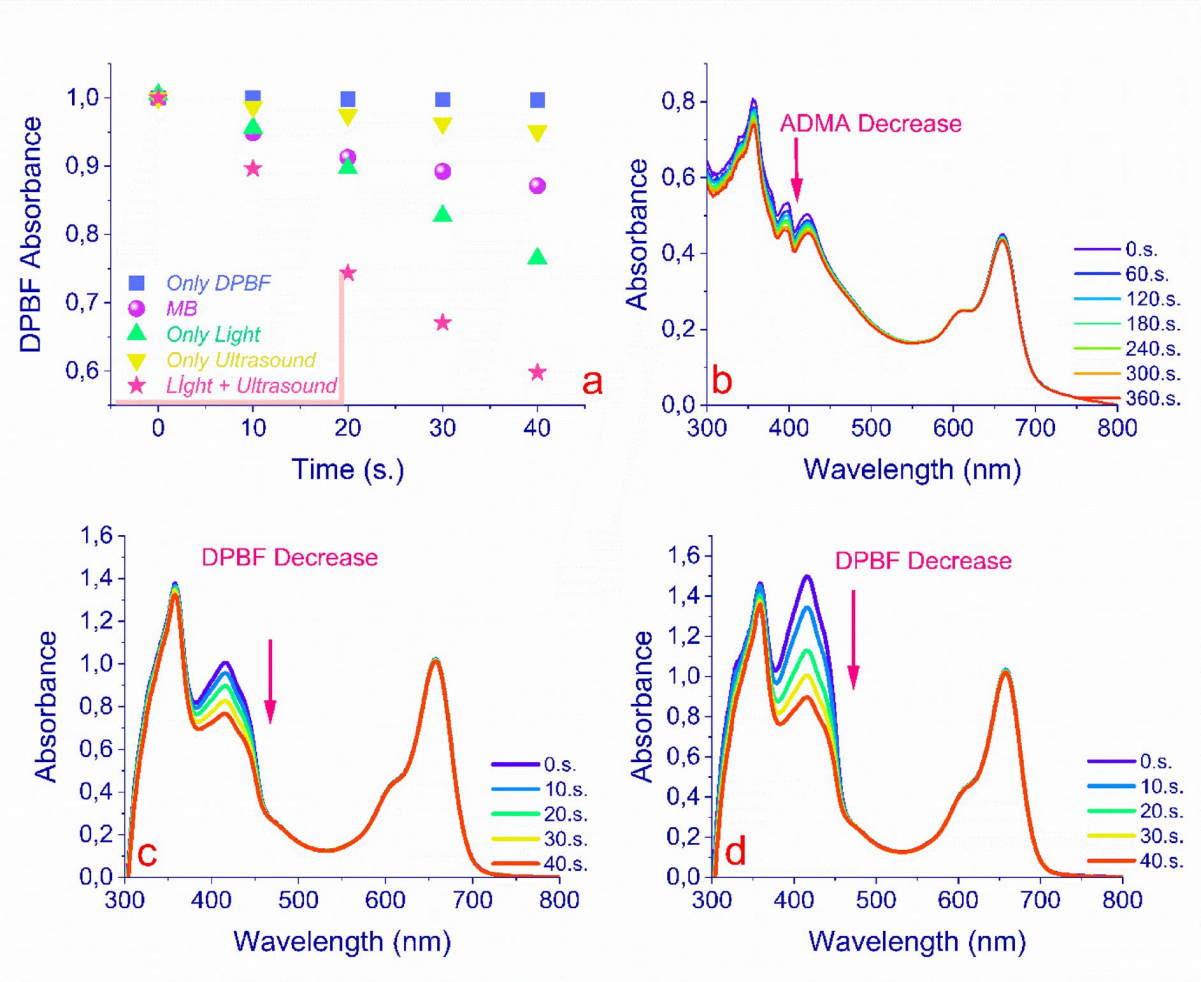
(**a**) Time-dependent decrease of absorbance at 417 nm by oxidation of DPBF with *Q-BD* under light and ultrasound + light, (**b**) Determination of singlet oxygen production for *Q-BD* in H_2_O via photochemical pathway, (**c**) Determination of singlet oxygen production for *Q-BD* in DMSO via photochemical pathway, (**d**) Determination of singlet oxygen production for *Q-BD* in DMSO via sono-photochemical pathway.

In this study, singlet oxygen generation was evaluated using the DPBF chemical trapping method, which is widely employed to detect ^1^O_2_ and therefore primarily reflects the Type II photodynamic pathway. The significant decrease in DPBF absorbance under light irradiation suggests efficient singlet oxygen production. Singlet oxygen quantum yield (Φ_Δ_) values are listed in Table 1. The obtained spectra and plot of the decrease in absorbance of DPBF against time are shown in Fig. 2 for *BD* and in Fig. 3 for *Q-BD.*

In photochemical calculations, *BD* exhibits a lower singlet oxygen production compared to *Q-BD*. This reduction is attributed to a ICT process induced by the presence of *N,N*-dimethyl groups in *BD*. Specifically, electron transfer occurs from the electron-rich dimethylamine groups to the electron-deficient BODIPY core, which in turn diminishes the efficiency of singlet oxygen generation. It was seen that after quaternization of *N,N*-dimethyl segments, singlet oxygen production for *Q-BD* was notable increased. The enhanced singlet oxygen production arose from the protonation of the amino groups in *Q-BD* can be attributed to the suppression of the intramolecular charge transfer process^63,64^.The water-soluble *Q-BD* demonstrates a 28% higher singlet oxygen production compared to the reference molecule, methylene blue (MB) (see Fig. 3a), which can be attributed to the presence of iodine atoms, thiophene moiety, and the suppression of the (ICT) process within its molecular structure.

In the sono-photochemical calculations, which is a combined application of these modalities (PDT and SDT), a significant synergistic effect was observed, leading to an enhanced production of singlet oxygen in both compounds. To assess the effect of ultrasound alone, a control experiment was performed using DPBF in the absence of the sensitizer, applying only ultrasound or ultrasound combined with light. These results confirmed that under the applied conditions, DPBF degradation without the photosensitizer was negligible, indicating that the observed enhancement arises from the interaction of the sensitizer with light and ultrasound (see Fig. S8). The observed enhancement is consistent with known effects of acoustic cavitation. The formation and collapse of cavitation bubbles generate localized high temperature and pressure, promoting radical formation and ROS production. Additionally, sonoluminescence generated during inertial cavitation can directly excite the sensitizer, enabling energy transfer to ground-state molecular oxygen and resulting in singlet oxygen formation via a Type II pathway. These cavitation-related processes are considered the most plausible contributors to the increased ROS production under ultrasound irradiation^18,25,65^.

#### Assessment of photo-resistance properties

**Fig. 4:**
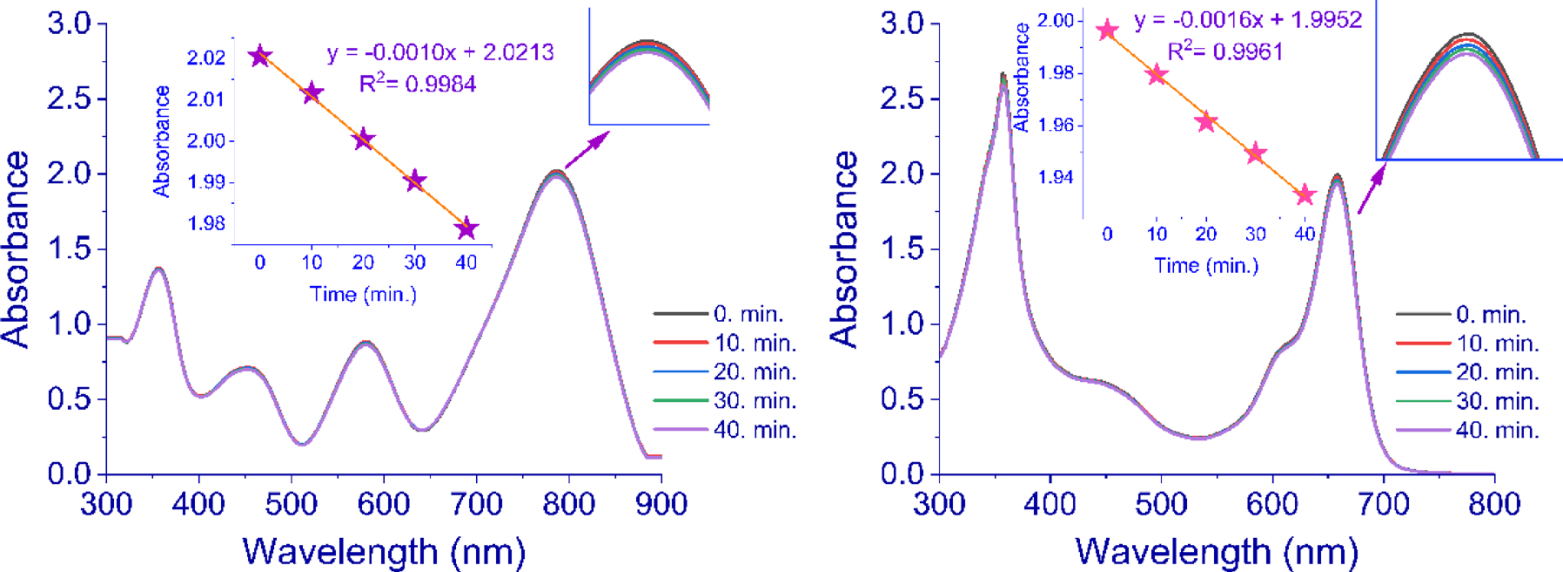
Determination of photostability of *BD* (left) and *Q-BD* (right) in DMSO via photochemical pathway.

Assessment of photostability is a critical prerequisite for the clinical application of potential sensitizers, as therapeutic efficacy relies on maintaining consistent drug concentration and activity throughout the application. To investigate this parameter, photodegradation studies were conducted by subjecting the sensitizers to high-intensity light irradiation at defined 10-min intervals. This approach enables the evaluation of structural and functional stability under certain conditions that simulate therapeutic light exposure. Figure 4 shows the spectral changes during the photostability determination experiment, and the results obtained were listed in Table 1. According to the results, both compounds showed moderate stability in an intense light.

### In vitro therapeutic evaluation of BODIPY-based sensitizers

**Fig. 5:**
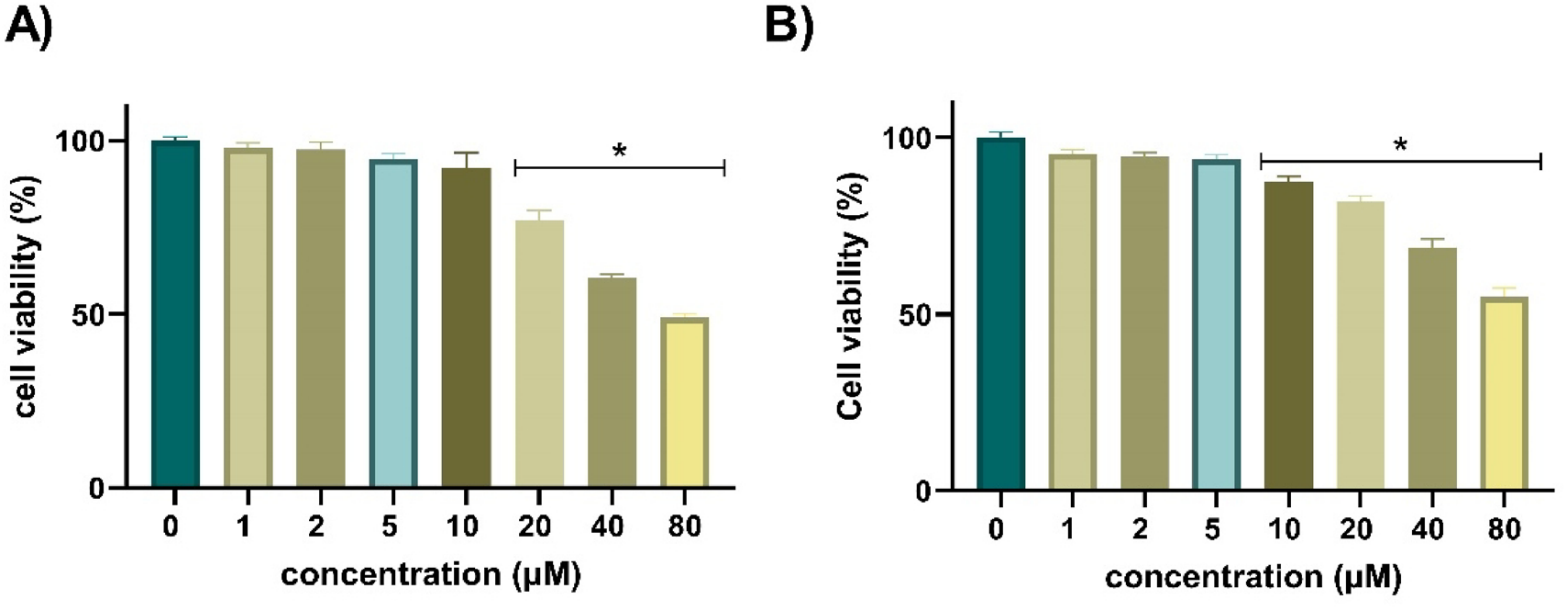
Cytotoxic effects of *BD* and *Q-BD* compounds on MDA-MB-231 cells. MTT cytotoxicity results of *BD* (**A**) and *Q-BD* (**B**) compounds in MDA-MB-231 cells after 24 h of incubation at concentrations ranging from 0 to 80 µM. Cell viability is expressed as a percentage relative to the untreated control group. Data are presented as mean ± SEM (*n* = 12). Statistical significance compared to the control group is indicated by *p < 0.05.

The cytotoxic effects of *BD* and *Q-BD* compounds on MDA-MB-231 cells were evaluated using the MTT assay across a concentration range of 0–80 µM. As shown in Fig. 5, both compounds exhibited a dose-dependent decrease in cell viability. However, concentrations up to 5 µM did not induce significant cytotoxicity compared to the control group, indicating their biocompatibility under dark conditions. Based on these findings, 5 µM was selected as the working concentration for subsequent SDT, PDT, and SPDT treatment experiments.

**Fig. 6:**
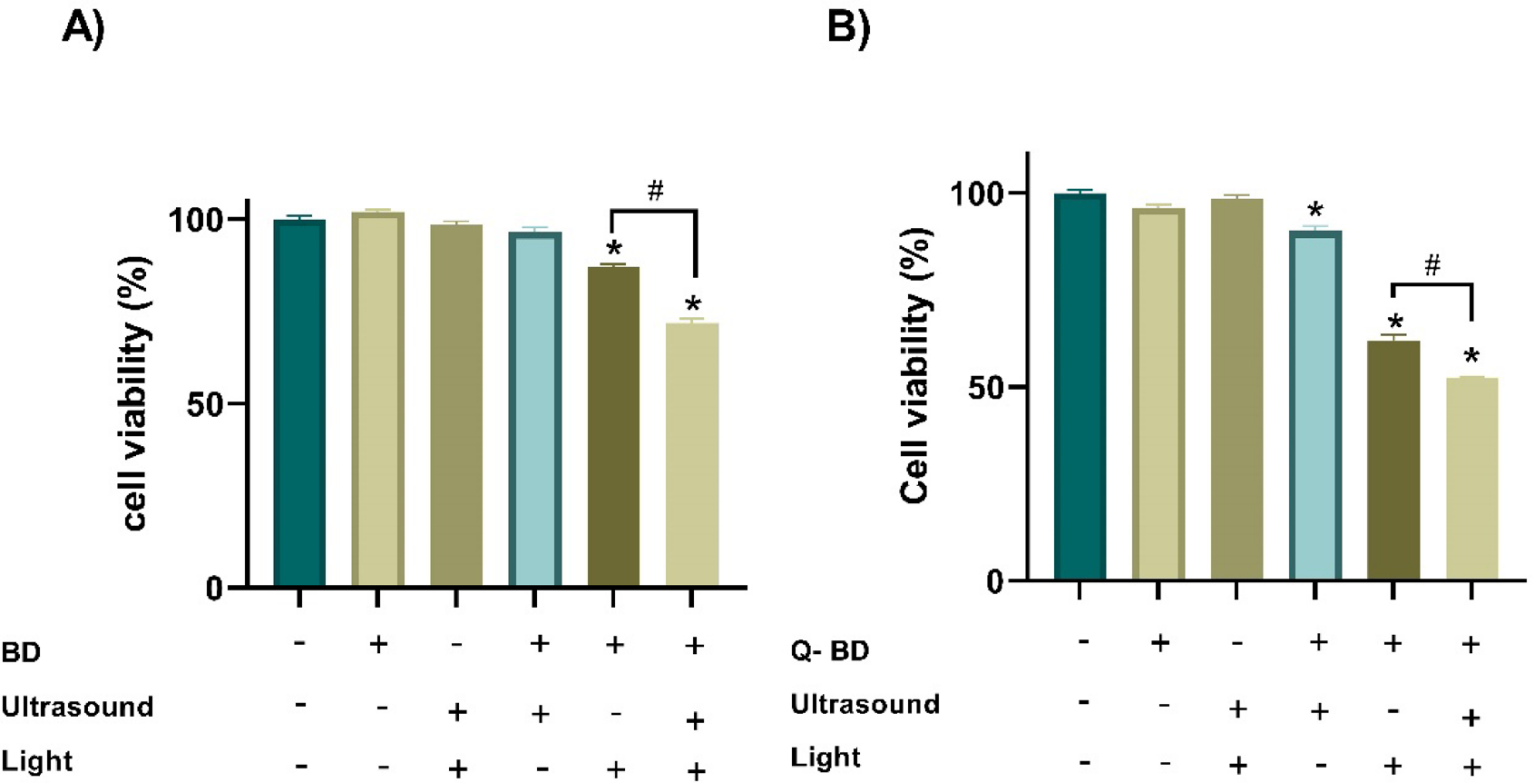
MTT-based cytotoxicity of SDT, PDT, and SPDT treatments in MDA-MB-231 cells. Cell viability was assessed 24 h post-treatment with *BD* (**A**) and *Q-BD* (**B**) compounds at 5 µM under SDT, PDT, and SPDT conditions. Data are presented as mean ± SEM (*n* = 12). Statistical significance was considered at *p* < 0.05, with comparisons to the control group indicated by * and significance between PDT and SPDT groups indicated by #.

The therapeutic efficacy of *BD* and *Q-BD* compounds under SDT, PDT, and SPDT treatment conditions was evaluated in MDA-MB-231 cells 24 h post-treatment using MTT and Annexin V-FITC/PI assays. As shown in Fig. 6A, B, PDT and SPDT applications significantly reduced cell viability compared to the untreated control groups. Among the treatments, SPDT exhibited the highest cytotoxic effect, indicating a synergistic effect of combined ultrasound and light exposure. Both *BD* and *Q-BD* compounds showed similar trends, although *Q-BD* induced slightly higher cell death in SPDT-treated cells.

To quantitatively evaluate the photocytotoxic efficacy of the compounds, IC_50_ values were calculated under both dark and SPDT conditions (Table 2). *Q-BD* exhibited an IC_50_ value of approximately 89.86 µM in the dark, which markedly decreased to 6.74 µM following SPDT treatment. This corresponds to a SPI of approximately 13.33, indicating a pronounced light-dependent cytotoxic effect and high therapeutic selectivity.

**Table 2:** IC_50_ values and phototherapeutic index of *BD* and *Q-BD* under dark and SPDT conditions.

Compound	IC_50_ Dark (µM)	IC_50_ SPDT (µM)	Sono-phototherapeutic index (SPI)
*BD*	71.62	8.57	8.35
*Q-BD*	89.86	6.74	13.33

In comparison, *BD* demonstrated an IC_50_ value of approximately 71.62 µM under dark conditions and 8.57 µM under SPDT, resulting in a SPI of approximately 8.35. The notably higher SPI value observed for *Q-BD* suggests superior photoselectivity and enhanced therapeutic efficiency under SPDT conditions.

**Fig. 7:**
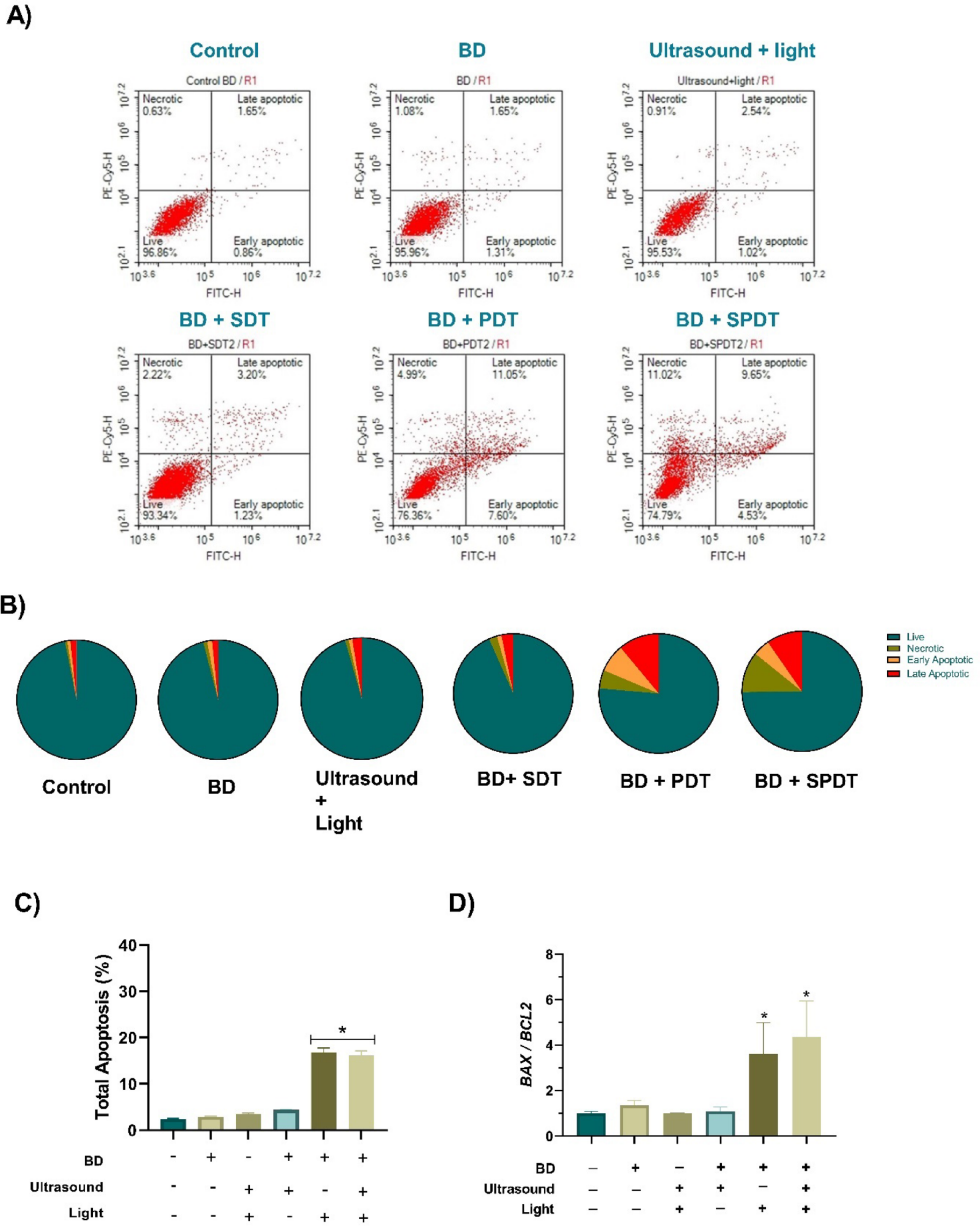
Apoptosis analysis following *BD*-mediated treatments. (**A**) Flow cytometry analysis using Annexin V-FITC/PI to evaluate cell death in MDA-MB-231 cells 24 h post-treatment. Experimental groups included Control, Ultrasound + Light, *BD*, *BD* + SDT, *BD* + PDT, *BD* + SPDT (**B**) Pie charts represent the distribution of live, necrotic, early apoptotic, and late apoptotic cells (%) across all treatment groups. (**C**) Total apoptosis (%) was calculated for each treatment condition. (**D**) BAX/BCL-2 mRNA expression levels are shown as bar graphs. Fold changes were normalized to β-actin and calculated relative to the control group. Data are expressed as mean ± SEM from three independent experiments, with qPCR performed in triplicate. Statistical significance was considered at *p* < 0.05, with comparisons to the control group indicated by * and significance between PDT and SPDT groups indicated by #.

**Fig. 8:**
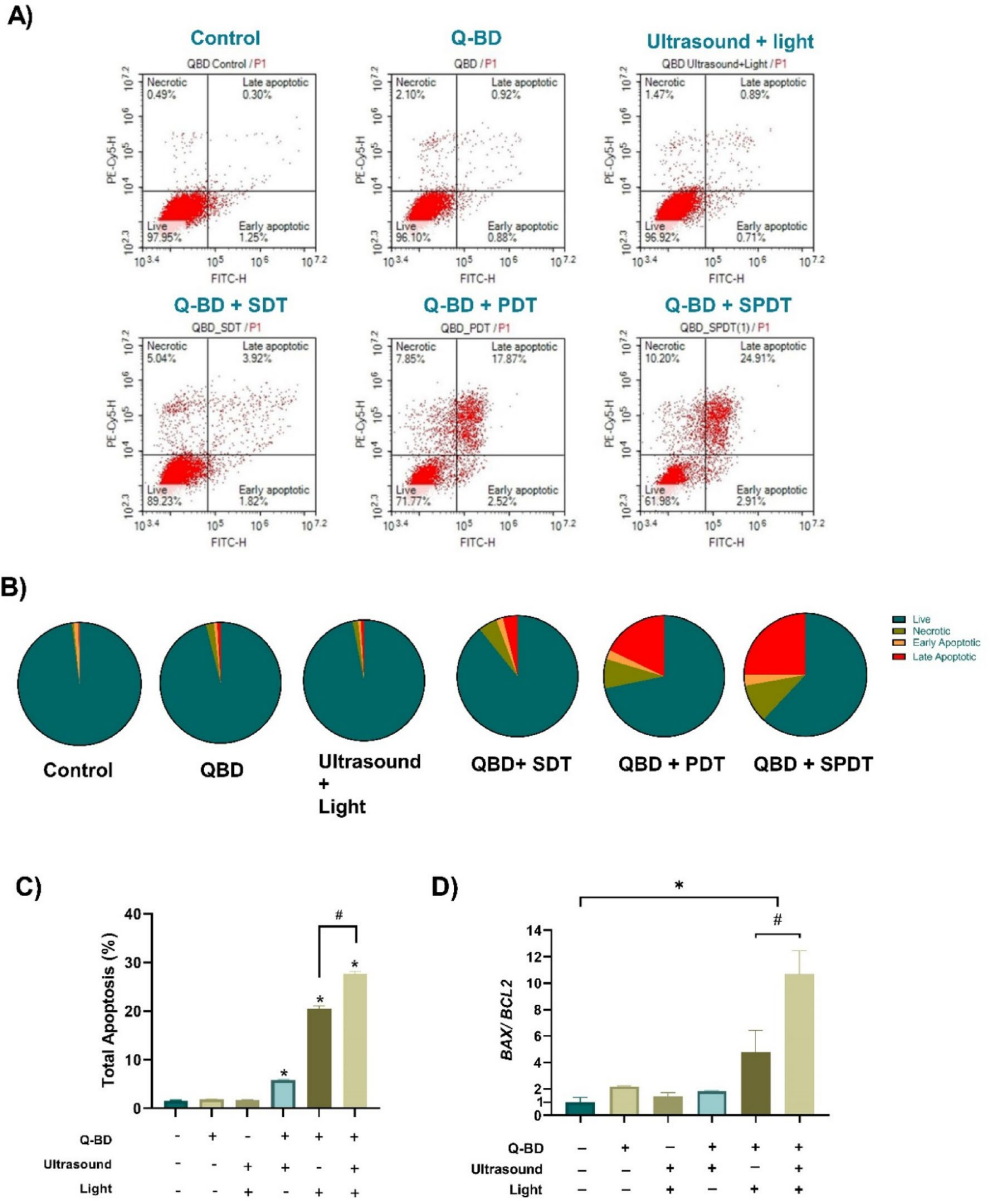
Apoptosis analysis following *Q-BD*-mediated treatments. (**A**) Flow cytometry analysis using Annexin V-FITC/PI to evaluate cell death in MDA-MB-231 cells 24 h post-treatment. Experimental groups included Control, Ultrasound + Light, *Q-BD*, *Q-BD* + SDT, *Q-BD* + PDT, *Q-BD* + SPDT. (**B**) Pie charts represent the distribution of live, necrotic, early apoptotic, and late apoptotic cells (%) across all treatment groups. (**C**) Total apoptosis (%) was calculated for each treatment condition. (**D**) BAX/BCL-2 mRNA expression levels are shown as bar graphs. Fold changes were normalized to β-actin and calculated relative to the control group. Data are expressed as mean ± SEM from three independent experiments, with qPCR performed in triplicate. Statistical significance was considered at *p* < 0.05, where * denotes comparisons versus the control group.

Representative flow cytometry plots for *BD*- and *Q-BD*-mediated treatments are shown in Figs. 7A and 8A, respectively, while the quantitative distributions of necrotic, early and late apoptotic cells are presented in Figs. 7B, C and 8B, C. The percentage of apoptotic cells markedly increased in the PDT, and SPDT groups compared to the control and compound-only treatments, with SPDT inducing the highest apoptotic rate, consistent with the observed decrease in cell viability. Furthermore, the BAX/BCL2 mRNA expression ratio was significantly elevated following treatment (Figs. 7D, 8D), indicating activation of the mitochondria-dependent apoptotic pathway^66,67^. This increase was most pronounced in the SPDT group, suggesting that the combined ultrasonic and photonic stimulation synergistically enhanced mitochondrial membrane permeabilization and caspase-mediated apoptotic signaling.

**Fig. 9:**
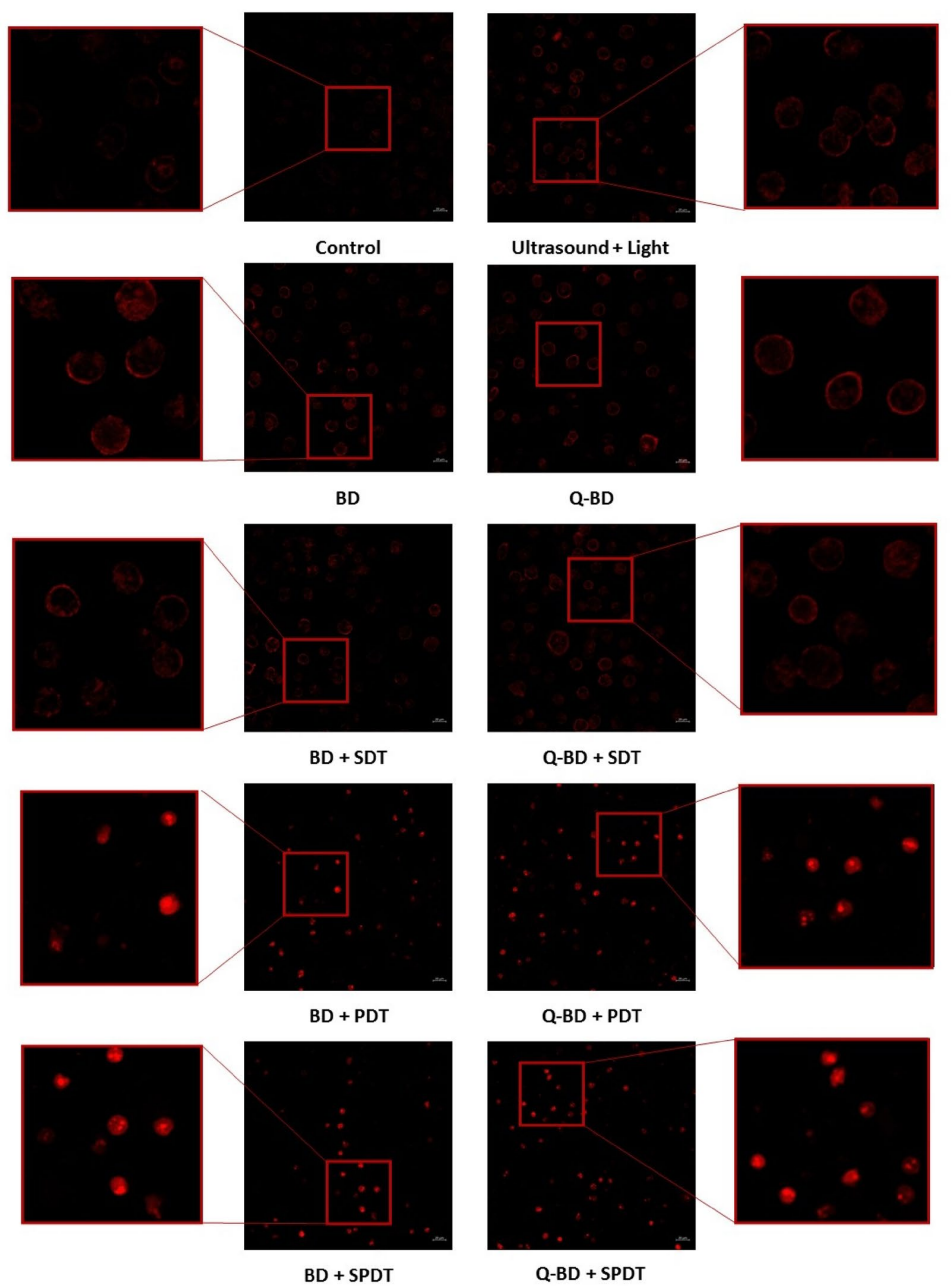
SPDT-induced intracellular ROS generation.

To further explore the mechanism underlying SPDT-induced apoptosis, intracellular ROS production and mitochondrial antioxidant responses were analyzed. As shown in Fig. 9, DHE staining followed by confocal microscopy revealed a marked increase in red fluorescence intensity in SPDT-treated cells compared to the control, ultrasound + light, and single-treatment groups. The fluorescence intensity maps demonstrated extensive ROS accumulation in both *BD*- and *Q-BD*-treated cells, particularly under SPDT conditions, whereas minimal signals were observed in control and compound-only groups.

Consistently, quantitative flow cytometric analysis of DHE fluorescence (Fig. S9) confirmed a significant increase in intracellular ROS levels in SPDT-treated groups compared to the control and other treatment modalities.

**Fig. 10:**
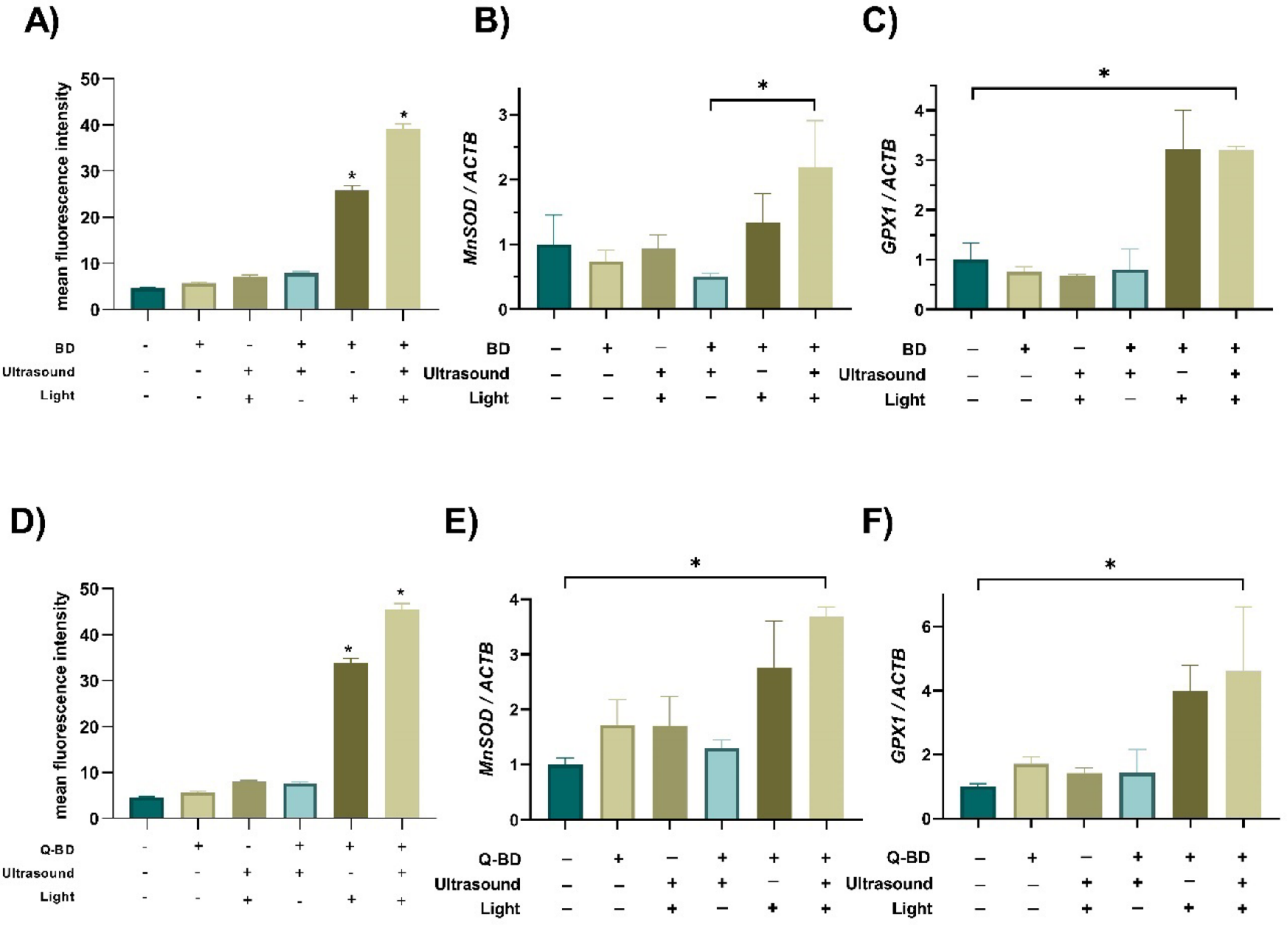
Quantitative analysis of ROS-associated mitochondrial antioxidant response. (**A**, **D**) Mean fluorescence intensity of DHE-stained MDA-MB-231 cells treated with *BD* and *Q-BD* compounds, respectively. Data are presented as mean ± SEM from three independent experiments. Data are expressed as mean ± SEM (*n* = 200 cells per group). (**B**, **E**) Relative mRNA expression of *MnSOD* normalized to *ACTB* in *BD*- and *Q-BD*-treated groups. (**C**, **F**) Relative mRNA expression of *GPX1* normalized to *ACTB* in *BD*- and *Q-BD*-treated groups. Statistical significance compared to the control group is indicated by *p < 0.05.

Quantitative analysis of mean fluorescence intensity confirmed these observations (Fig. 10A, D), showing a significant elevation in ROS levels in the SPDT group relative to all other treatments (*p* < 0.05). To investigate whether this ROS accumulation affected mitochondrial antioxidant defenses, *MnSOD* and *GPX1* mRNA expressions were quantified. As shown in Fig. 10B, C, E, F, both *MnSOD*/*ACTB* and *GPX1*/*ACTB* ratios were significantly increased following SPDT, suggesting an adaptive mitochondrial antioxidant response triggered by elevated ROS production^53,68^.

Representative confocal microscopy images (20 ×) of DHE-stained MDA-MB-231 cells after treatments with *BD* and *Q-BD* compounds under control, ultrasound + light, and combined SDT, PDT, and SPDT conditions. Zoomed regions highlight localized ROS accumulation.

Overall, these findings demonstrate the enhanced therapeutic potential of SPDT over single-modality treatments in MDA-MB-231 cells. The observed increase in apoptosis and reduction in cell viability highlights the synergistic effect of ultrasound-mediated cavitation and light-triggered ROS generation, supporting the rationale for combined sono-photodynamic therapy as a promising anticancer strategy using *BD-* and *Q-BD* compounds; however, the lack of in vivo validation, absence of targeted delivery strategies, and the need for comprehensive analyses such as proteomic or transcriptomic profiling to elucidate global cellular responses represent important limitations that should be addressed in future studies.

### Molecular docking results

Molecular docking analyses of the newly synthesized *BD* and *Q-BD* compounds, along with cisplatin as a positive control, with the target protein EGFR were performed using AutoDock Tools 1.5.6. The calculated binding energy values for *BD*, *Q-BD*, and cisplatin were − 10.47 kcal/mol, − 11.07 kcal/mol, and − 3.56 kcal/mol, respectively. Since more negative binding energy values correspond to higher binding affinity, these findings indicate that both newly synthesized compounds interact more strongly with EGFR than cisplatin, which is currently used as a clinical breast cancer drug.

**Fig. 11:**
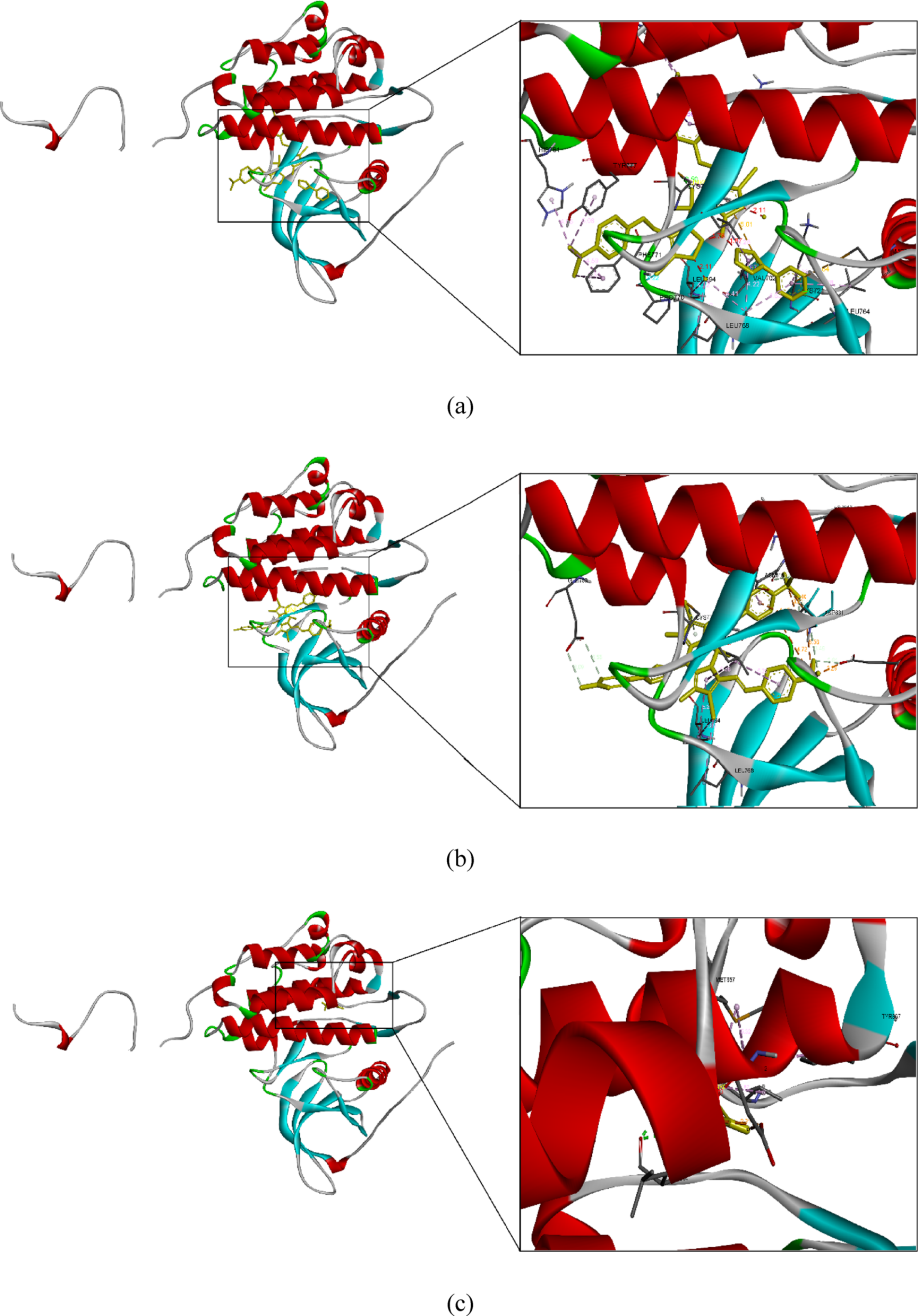
Interaction diagrams of (**a**) *BD*, (**b**) *Q-BD*, (**c**) Cisplatin with EGFR.

A detailed interaction profile revealed that *BD* forms key contacts with several EGFR residues (Fig. 11a). Specifically, a conventional hydrogen bond was formed with CYS773 at a distance of 3.503 Å. In addition, hydrophobic interactions, including alkyl, π-alkyl, and π-sigma types, predominantly occurred with active-site residues such as LEU694, ALA719, LEU764, LEU768, PHE771, and LEU820 (Table 3). These interactions collectively suggest robust molecular recognition, primarily mediated by hydrogen bonding and hydrophobic contacts.

**Table 3:** Binding energy values and key binding interactions of *BD*, *Q-BD* and cisplatin with EGFR.

Ligand	Distance (Å)	Interaction type	Residue
*BD*	3.503	Conventional hydrogen bond	CYS73
	3.669	π-Sigma	VAL702
	3.584	π-Sigma	LEU775
	5.640	π-Sulfur	MET742
	4.414	Alkyl	ALA719
	4.221	Alkyl	LEU694
	5.121	Alkyl	LEU768
	4.260	Alkyl	LEU775
	4.586	Alkyl	LYS889
	4.594	π-Alkyl	PHE771
	5.081	π-Alkyl	TYR777
	4.064	π-Alkyl	HIS781
	5.226	π-Alkyl	CYS773
	4.217	π-Alkyl	ALA719
	5.283	π-Alkyl	LEU820
	4.626	π-Alkyl	ALA719
	3.894	π-Alkyl	LYS721
	5.278	π-Alkyl	LEU764
	4.749	π-Alkyl	ARG817
*Q-BD*	3.294	Attractive charge	GLU738
	4.718	Attractive charge	ASP831
	5.563	Attractive charge	ASP831
	4.250	Attractive charge	ASP813
	3.813	Attractive charge	ASP831
	3.398	Attractive charge	ASP831
	3.584	Carbon hydrogen bond	GLU780
	3.085	Carbon hydrogen bond	GLU780
	2.607	Carbon hydrogen bond	GLU738
	3.494	Carbon hydrogen bond	ASP831
	2.886	Carbon hydrogen bond	ASP813
	3.440	Carbon hydrogen bond	ASP818
	3.402	Carbon hydrogen bond	ASP831
	2.910	Carbon hydrogen bond	ASP831
	4.154	π-Anion	ASP831
	3.942	π-donor hydrogen bond	CYS773
	4.885	Alkyl	LEU694
	5.002	Alkyl	LEU768
	5.278	π-Alkyl	LEU694
	5.383	π-Alkyl	LEU820
	5.386	π-Alkyl	LEU820
	5.050	π-Alkyl	ARG817
Cisplatin	2.310	Attractive charge	ASP872
	2.144	Attractive charge	ASP872
	2.273	Conventional hydrogen bond	LEU814
	2.291	Conventional hydrogen bond	LEU814
	5.321	Alkyl	MET857
	4.873	Alkyl	VAL852
	4.415	Alkyl	PRO853
	4.406	Alkyl	MET857
	5.124	π-Alkyl	TYR867

Similarly, docking analysis of *Q-BD* with EGFR showed the formation of eight carbon–hydrogen bonds with EGFR residues, predominantly involving the active-site residue ASP831 (Fig. 11b). Moreover, alkyl and π-alkyl interactions were mainly observed with active-site residues LEU694, LEU768, and LEU820. The abundance of these interactions likely accounts for *Q-BD*’s high binding affinity toward EGFR.

In contrast, cisplatin did not interact with the active site of EGFR and exhibited fewer contacts than the two newly synthesized compounds (Fig. 11c). This interaction profile aligns with its considerably lower binding affinity, as reflected by its less negative binding energy value.

Finally, to validate the accuracy of the molecular docking procedure, a self-docking experiment was performed. The resulting RMSD value (1.03 Å) was well below the literature-defined upper threshold of 2 Å^69^, thereby confirming the reliability of the docking methodology.

These docking results collectively highlight the superior binding affinity and interaction profiles of the newly synthesized *BD* and *Q-BD* compounds toward EGFR, in stark contrast to cisplatin.

## Conclusion

This study demonstrates the promising potential of newly synthesized BODIPY derivatives as effective sensitizers in SPDT. Singlet oxygen quantum yields of *BD* and *Q-BD* were determined both photochemically and sono-photochemically. In both techniques, *Q-BD* showed better performance demonstrating an efficient formation of singlet oxygen when simultaneously activated by light and ultrasound compared to *BD*. In vitro analyses in MDA-MB-231 triple-negative breast cancer cells revealed that SPDT induced a significant reduction in cell viability and a marked increase in apoptotic cell death compared to individual PDT or SDT treatments. Enhanced intracellular ROS production and the upregulation of antioxidant enzymes (*MnSOD* and *GPX1*) confirmed that oxidative stress played a major role in the cytotoxic response. These findings highlight the synergistic therapeutic advantage of combining light and ultrasound and suggest that BODIPY-based compounds could serve as dual-acting sensitizers for future SPDT applications in resistant cancer types such as triple-negative breast cancer. Molecular docking analyses revealed that both newly synthesized compounds exhibited substantially higher binding affinity toward EGFR compared to cisplatin, as evidenced by more negative binding energy values and extensive interactions with key active-site residues. Considering all experiments and data, both *BD* and *Q-BD* can be regarded as promising sensitizer agents in SPDT for related cancer treatments.

## Supplementary Information

Below is the link to the electronic supplementary material.

Supplementary Material 1

## Data Availability

All data generated and analyzed during the current study were produced specifically for this research and are available from the corresponding author upon reasonable request.
