# Supplementary material for: Light and ultrasound activated precision: a novel water-soluble BODIPY-mediated sono-photosensitizer in SPDT for breast cancer treatment
Source: Sci Rep. 2026 Apr 16;16:17637. doi: 10.1038/s41598-026-48642-9 (PMC13243447; doi:10.1038/s41598-026-48642-9)
Supplement: Supplementary file 1 — Supplementary Material 1 [file 41598_2026_48642_MOESM1_ESM.docx]

***Light and Ultrasound Activated Precision: A Novel Water-Soluble BODIPY-Mediated Sono-Photosensitizer in SPDT for Breast Cancer Treatment***

**Ceren Can Karanlık^a,c^, Ayşegül Türkkol^b^, Gürkan Karanlık^a,c^, Umut Kerem Kolaç^d^, Şerife Gökçe Çalışkan^e^ , Mehmet Dinçer Bilgin^b^, Ali Erdoğmuş^a,c*^**

^a^Department of Chemistry, Faculty of Arts and Science, Yildiz Technical University, Istanbul, 34220, Turkey

^b^Department of Biophysics, Faculty of Medicine, Aydin Adnan Menderes University, Aydin, 09010, Turkey

^c^Health Biotechnology Joint Research and Application Center of Excellence, 34220, Istanbul, Turkey

^d^Department of Medical Biology. Faculty of Medicine, Aydin Adnan Menderes University, Aydin, 09010, Turkey

^e^Department of Physics, Faculty of Sciences, Aydın Adnan Menderes University, 09010 Aydın, Turkey

*Corresponding author; [aerdog@yildiz.edu.tr](mailto:aerdog@yildiz.edu.tr) (A. ERDOGMUS) Tel: + 90 212 383 41 71

**SUPPORTING INFORMATION**

**Table of Contents**

1. **Materials……………………………………………………………..………..…..…S2**
2. **Instrumentation………………………………………..……………………….…....S2**
3. **The Parameters for Fluorescence Quantum Yield (Φ_F_)…………………….....….S2**
4. **The Parameters for Singlet Oxygen Quantum Yield (Φ_Δ_)………...……..…....….S3**
5. **The Parameters for Photodegradation Quantum Yield (Φ_d_)…………….……….S4**
6. **Spectral Data (^1^H-NMR, FT-IR and Mass Spectra) ……………….…..………...S4**
7. **List of primers and their sequences………………………………………………..S8**

**1. Materials**

The chemical substances and solvents used in experimental studies, 5-bromothiophene-2-carbaldehyde, 2,4-dimethylpyrrole, trifluoroacetic acid (TFA), boron trifluoride diethyl etherate (BF_3_.OEt_2_), triethylamine (Et_3_N), 4-pyridinylboronic acid, Pd(PPh_3_)_4_, iodine (I_2_), iodic acid (HIO_3_), 4-(Dimethylamino)benzaldehyde, chloroform (CHCl_3_), hexane, ethyl acetate, acetone, dry toluene, piperidine, glacial acetic acid, K_2_CO_3_, sodium sulfate (Na_2_SO_4_), methanol (MetOH), dimethyl sulfoxide (DMSO), N,N-dimethylformamide (DMF), 1,3-diphenylisobenzofuran (DPBF) and 9,10-anthracenediyl-bis(methylene)dimalonic acid (ADMA), Methylene Blue (MB) were supplied commercially from Sigma-Aldrich and used without further purification.

Reactions were monitored by thin layer chromatography (TLC) on aluminium plates coated with silica gel 60 F254 Merck. Column chromatography on silica gel was performed over Merck silica gel 60 (pore size 60 Å, 0.040-0.063 mm particle size).

**2. Instrumentation**

Absorption spectra in the UV-Visible region were obtained with a Shimadzu 2001 UV spectrophotometer. Fluorescence spectra were measured using a Varian Eclipse spectrofluorometer using 1 cm path length cuvettes at room temperature.

^1^H-NMR spectra were recorded at room temperature in deuterated DMSO solution on a Bruker spectrometer with tetramethylsilane as internal standard. Photo-irradiation was measured using a General Electric quartz line lamp (300W). A 600 nm glass cut off filter (Schott) and a water filter were used to filter off ultraviolet and infrared radiation respectively. Interference filters (Intor, 670 nm with a bandwidth of 40 nm for *Q-BD, 750 nm with a bandwidth of 40 nm for BD*) were additionally placed in the light path before the sample. Light intensities were measured with a POWER MAX5100 (Mol electron detector incorporated) power meter.

**3. The Parameters for Fluorescence Quantum Yield (Φ_F_)**

Fluorescence quantum yields of *BD* and *Q-BD* were measured in DMSO which was calculated by using fluorescein as the reference quantum yield (Φ_F(std)_ = 0.79 in EtOH ) [[1](#_ENREF_1)].

To determine the emission properties and fluorescence quantum yileds of *BD and Q-BD*, solution were prepared in DMSO with concentration 1.8 × 10^-5^ M for *BD* and 8.2 × 10⁻^6^ M for *Q-BD*. The fluorescence quantum yields (Φ_F_) were determined by the comparative method (Eq. (1)).

(1)

where F and F_Std_ are the areas under the fluorescence emission curves of *BD* and *Q-BD* and the standard, respectively. A and A_Std_ are the respective absorbances of *BD* and *Q-BD* and the standard at the excitation wavelengths, respectively. η and η_std_ are there refractive indexes of solvents (η_EtOH_: 1.361, η_DMSO_: 1.480) [[2](#_ENREF_2)] used for sample and standard, respectively. There refractive indices (η) of the solvents were employed in calculating the fluorescence quantum yield in different solvents.

**4. The Parameters for Singlet Oxygen Quantum Yield (Φ_Δ_)**

To determine the singlet oxygen production capacities of *compounds*, 1,3-diphenylisobenzofuran (DPBF), also ADMA for *Q-BD*, *BD* and *Q-BD* solutions were prepared in DMSO and kept in a dim light environment. A sample of 2 mL was prepared (DPBF: *BD* and *Q-BD*, 1:1 (v/v)) and added to the cuvette and exposed to light (intensity of 7.05 × 10^15^ photons s^-1^ cm^-2^) at 10 s intervals at room temperature in dark, and the decrease in absorption of DPBF at 417 nm was recorded with using UV–Vis spectroscopy.

Singlet oxygen efficiency was determined in the air (no oxygen bubbled) using the relative method (Eq. **2**) with methylene blue (MB) as standard, 1,3-diphenylisobenzofuran (DPBF) as chemical quencher for singlet oxygen.

$\Phi_{\Delta} = \Phi_{\Delta}^{std} \frac{m_{sample}}{m_{std}}\times\frac{F_{std}}{F_{sample}}$ **2**

Where $\Phi_{\Delta}^{std}$ is the singlet oxygen quantum yield for the standard ($\Phi_{\Delta MB}$= 0.52 in DMSO). m_sample_ and m_Std_ represent for slope of the plot of the absorbance of DPBF (at 417 nm) versus irradiation time. F can be calculated by F: 1-10^-OD^; OD and F stand for optical density and absorbance correction factor, respectively [[3](#_ENREF_3)] (OD: absorbance value at the wavelength at which it is excited).

For sono-photochemical studies, the cuvette containing solution is submerged in the rectangular water bath filled with water for five seconds before the light irradiation. The sample (*BD* and *Q-BD* +DPBF) was monitored after each 10 s irradiation (5 s by light intensity of 7.05 × 10^15^ photons s^-1^ cm^-2^ and 5 s by ultrasound at a frequency of 35 kHz). The ultrasonic bath generates the ultrasound by means of intensive cavitation bubbles. The acoustic excitation is a continuous wave at frequency of 35 kHz and the power of 320 watt [[4](#_ENREF_4)].

**5. The Parameters for Photodegradation Quantum Yields**

Photodegradation quantum yields of *BD* and *Q-BD* were determined using Eq. **3**,

 (3)

where “C_0_” and “C_t_” are the sample concentrations before and after irradiation respectively, “V” is the reaction volume, “N_A_” is the Avogadro’s constant, “S” is the irradiated cell area, “t” is the irradiation time, “I_abs_” is the overlap integral of the radiation source light intensity and the absorption of the sample. A light intensity of 2.42 x 10^16^ photons s^-1^ cm^-2^ and/or ultrasound at a frequency of 35 kHz was employed to determine photodegradation. The degradation of max. Q band was monitored after each 20 minute irradiation.

**6. Spectral Data (^1^H-NMR, FT-IR and Mass Spectra)**

**Figure S1.** ^1^H-NMR spectrum of *BD*.





**Figure S2.** FT-IR spectrum of *BD*.

**Figure S3.** MALDI-TOF(MS) spectrum of *BD*.

**Figure S4.** ^1^H-NMR spectrum of *Q-BD*.





**Figure S5.** FT-IR spectrum of *Q-BD*.

**Figure S6.** MALDI-TOF(MS) spectrum of Q-*BD*.

**Table S1.** List of primers and their sequences.

| **Gene** | **Forward (5'-3')** | **Reverse (5'-3')** |
| --- | --- | --- |
| *BCL2* | CATGTGTGTGGAGAGCGTCAA | GCCGGTTCAGG TACTCAGTCA |
| *BAX* | TCGCCCTTTTCTACTTTGCC | AGTCTCACCCAACCACCCT |
| *MnSOD* | AAGGGAGATGTTACAGCCCAGATA | TCCAGAAAATGCTATGATT |
| *GPX1* | GGGACTACACCCAGATGAA | TCTCTTCGTTCTTGGCGTTC |
| *Β-ACTIN* | AACTGGGACGACATGGAGAA | GAAGGTCTCAAACATGATCTGG |

**
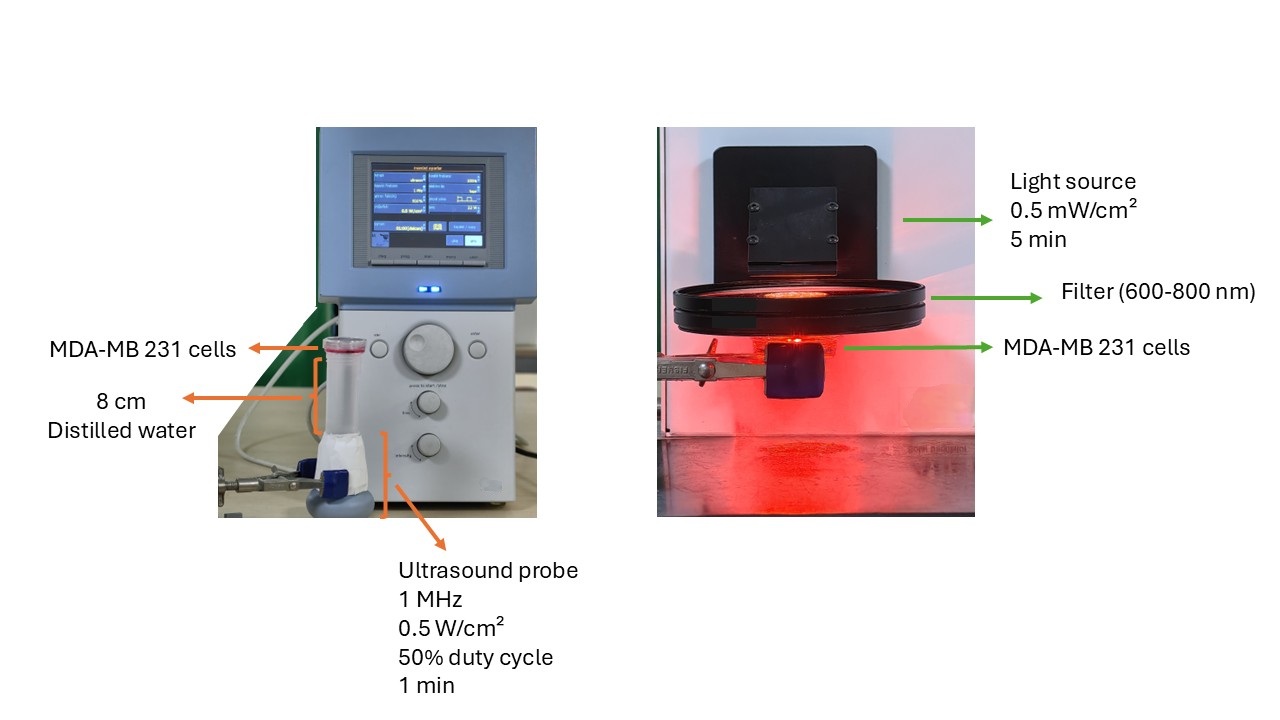
**

**Figure S7.** Experimental setup used for sono-photodynamic treatment.


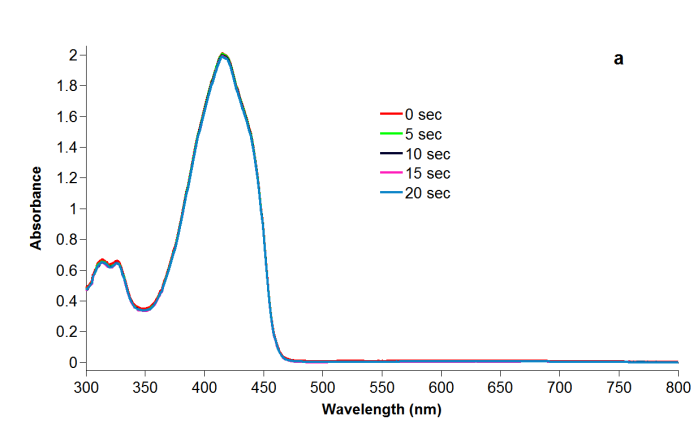

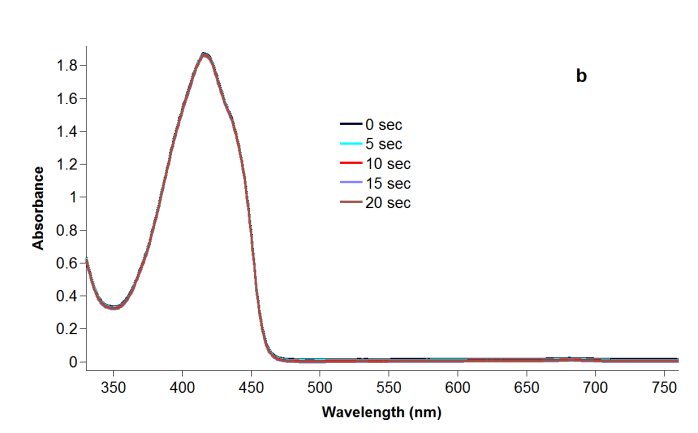


**Figure S8**. Spectral changes of only DPBF during the determination of singlet oxygen generation under (a) sonochemical and (b) sono-photochemical irradiation conditions.

**
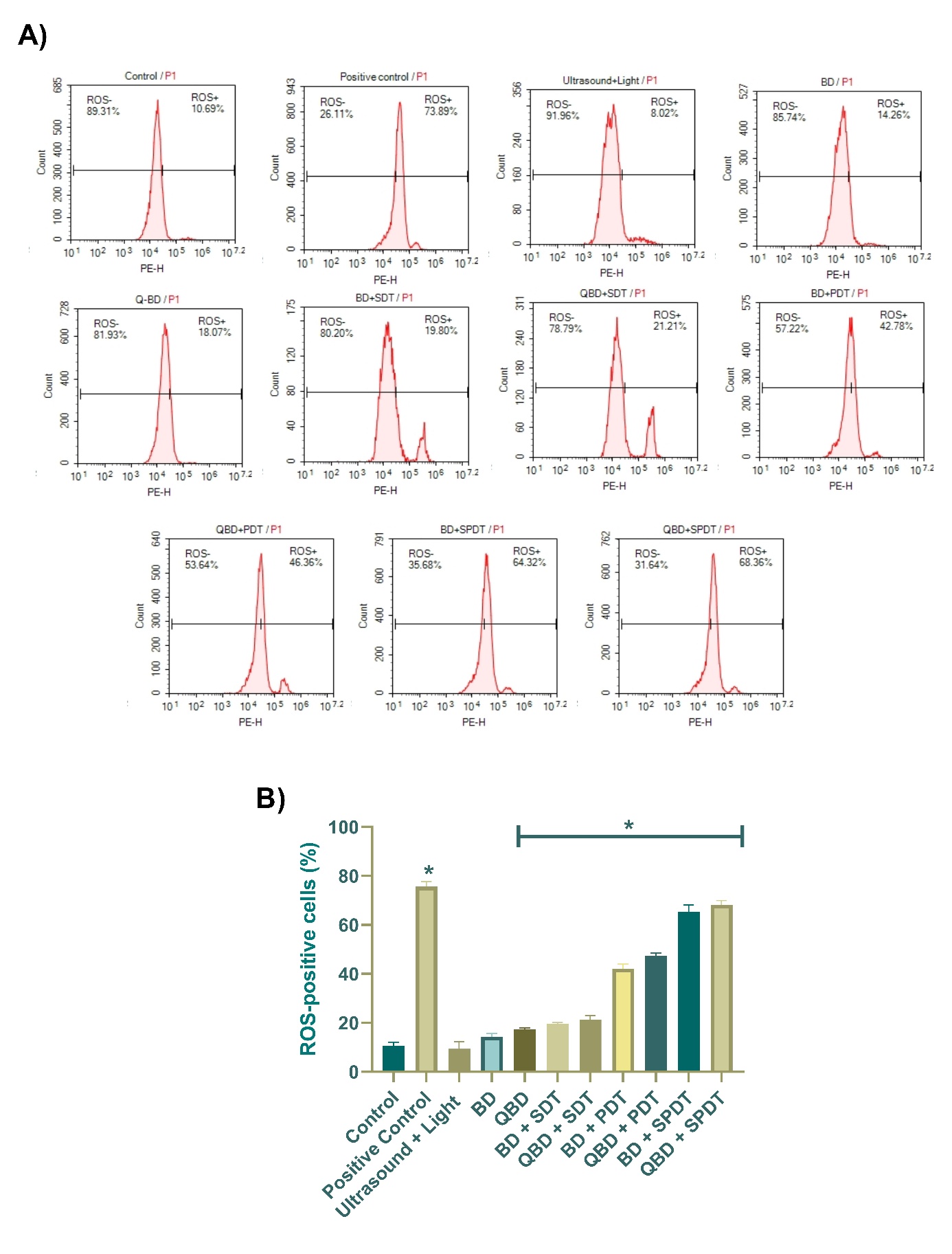
**

**Figure S9.** Flow cytometric analysis of intracellular ROS generation using DHE staining.

(A) Representative flow cytometry histograms showing DHE fluorescence intensity in control and treated groups.

(B) Quantitative analysis of ROS-positive cell populations expressed as the percentage of DHE-positive cells. Data are presented as mean ± SEM from at least three independent experiments. *p < 0.05 compared with the untreated control group.

**References**

[1] Çınar HŞ, Özçelik Ş, Kaya K, Kutlu ÖD, Erdoğmuş A, Gül A. Synthesis and photophysical properties of monomeric and dimeric halogenated aza-BODIPYs. Journal of Molecular Structure. 2020;1200:127108.

[2] Sen P, Atmaca GY, Erdoğmuş A, Dege N, Genç H, Atalay Y, et al. The synthesis, characterization, crystal structure and photophysical properties of a new meso-BODIPY substituted phthalonitrile. Journal of fluorescence. 2015;25:1225-34.

[3] Zhu J, Zou J, Zhang J, Sun Y, Dong X, Zhang Q. An anthracene functionalized BODIPY derivative with singlet oxygen storage ability for photothermal and continuous photodynamic synergistic therapy. Journal of Materials Chemistry B. 2019;7(20):3303-9.

[4] Karanlık CC, Atmaca GY, Erdoğmuş A. Comparison of singlet oxygen production of ethyl vanillin substituted silicon phthalocyanine using sonophotodynamic and photodynamic methods. Journal of Molecular Structure. 2023;1274:134498.
